# Digital Transformation of Medical Services in Romania: Does the Healthcare System Meet the Current Needs of Patients?

**DOI:** 10.3390/healthcare13202549

**Published:** 2025-10-10

**Authors:** Ioana-Marcela Păcuraru, Ancuța Năstac, Andreea Zamfir, Ștefan Sebastian Busnatu, Octavian Andronic, Andrada-Raluca Artamonov

**Affiliations:** 1 Faculty of Management, Bucharest University of Economic Studies, 010374 Bucharest, Romania; 2 Innovation and e-Health Center, Carol Davila University of Medicine and Pharmacy, 030167 Bucharest, Romania

**Keywords:** medical digitalization, quality of care, patient satisfaction

## Abstract

**Background**: The digitalization of medical services is promoted as a solution for improving access, quality, and efficiency within healthcare systems. In this context, the study investigates the extent to which digitalization in Romania meets the current needs of patients through a convergent analysis of user perceptions and managerial perspectives. Based on the specialized literature, the research tests two hypotheses: (H1) the implementation of digital technologies significantly contributes to improving the quality of medical services and operational efficiency; (H2) digitalization has a positive impact on patient satisfaction by facilitating access to care and improving communication with medical personnel. **Methods**: The study adopted methodology is cross-sectional and mixed, including an online mixed-methods questionnaire for patients, distributed between 6 and 14 May 2025, and a qualitative questionnaire with open-ended questions distributed via e-mail to managers from public hospitals through The Administration of Hospitals and Medical Services of Bucharest, between 3 and 24 March 2025. **Results**: In total, 125 patients and 15 hospital managers participated in the study. Statistical analysis (χ^2^, ordinal regression) and data triangulation highlight a predominantly positive, yet heterogeneous, patient perception of digitalization, with Hypothesis H1 only partially supported (weak, inconsistent, and in some cases negative associations between technology use and perceived service quality). By contrast, H2 was robustly validated, with patient satisfaction strongly linked to tangible benefits, particularly easier access and online appointment scheduling. However, use remains limited to administrative functions, while advanced technologies such as telemedicine or electronic health records are poorly adopted. From an institutional perspective, hospitals predominantly use IT systems for internal purposes, without real patient access to their own data, no interoperability between medical units, and marginal implementation of telemedicine. This reveals a significant gap between user perception and organizational realities, emphasizing the lack of a patient-oriented digital infrastructure. **Conclusions**: The results highlight the potential of digitalization to enhance patient experience and service efficiency, while also pointing out structural limitations that hinder the full realization of this potential. Patient satisfaction is strongly associated with tangible benefits, particularly easier access and online scheduling, whereas the effect on perceived quality is weaker and sometimes inconsistent. There are significant disparities in digitalization levels between healthcare providers, perceived by patients as public–private differences, and gaps among public hospitals are also confirmed by managerial data. These findings suggest that a successful digital transformation of the medical system in Romania must address both technological infrastructure gaps and organizational barriers, within a coordinated national strategy that ensures interoperability, patient-centered design, and sustainable implementation.

## 1. Introduction

The digital transformation of healthcare represents a great leap in the history of medicine, fundamentally reshaping how services are delivered, managed, and experienced. Since the earliest days of medicine, technological innovation has continuously influenced diagnostic, preventive, therapeutic, and rehabilitative practices, resulting in increasingly complex health systems with evolving roles for patients, professionals, and regulators [1]. In recent decades, the integration of digital technologies has become a central and influential process, already exerting substantial impact on healthcare systems and expected to further revolutionize care delivery in the future [2].

Such transformation involves the systematic introduction of new information and communication technologies and the corresponding redesign of processes within the sector. This process includes both healthcare-specific digitalization and broader societal trends, leading to significant changes in health technologies and care delivery [1,3]. The scope of digitalization is extensive, encompassing electronic health records (EHRs), telemedicine, wearable devices, artificial intelligence (AI), and big data, all of which are actively shaping the structure, culture, professions, treatments, and outcomes of health systems [3,4].

Despite its promise, the complexity and breadth of digital transformation present significant challenges, including confusion over terminology and concepts. The multidisciplinary nature of digital health, spanning legal, technical, organizational, and cultural domains, adds further complexity to its evaluation and implementation [1,2]. Legal issues such as privacy, cross-border data exchange, and interoperability must be addressed, while technical aspects like scalability, stability, and compatibility are critical for effective adoption [5]. Historically, healthcare has lagged behind other industries in digital innovation, partly due to regulatory constraints and the inherent complexity of healthcare delivery [6,7]. However, advances in IT, policy reforms, and increased investment have accelerated digital transformation, especially in response to the COVID-19 pandemic, which acted as a catalyst for rapid technology adoption and highlighted the value of digital solutions in crisis settings [2,3,5,8]. 

At the policy level, the European Union (EU) and national governments have made digital health a strategic priority, aiming to enhance care quality, efficiency, equity, affordability, and accessibility through innovation [1,6]. Initiatives such as the EU’s strategic plans and Australia’s nationwide My Health Record illustrate the global momentum toward integrated, patient-centered digital health systems. The success of digital transformation depends on the engagement and collaboration of diverse stakeholders, including end-users, developers, providers, and policymakers, as well as a nuanced understanding of both healthcare and digital domains [1,5]. In Romania specifically, digital transformation of the healthcare system is impeded by several key obstacles, including structural issues, inadequate funding, staffing shortages, outdated infrastructure, and low public trust [9]. According to the latest review by the Organisation for Economic Co-operation and Development (OECD), Romania currently faces significant challenges in healthcare digitalization, ranking 26th out of 27 EU countries in the Digital Economy and Society Index (DESI) for digital public services in health. The healthcare system is characterized by fragmented digital infrastructure, limited interoperability between healthcare institutions, and insufficient investment in digital health technologies. While government initiatives such as the National Electronic Health Record System and e-prescription systems are in development, their implementation remains limited and inconsistent across healthcare providers. This digital gap, combined with low digital literacy among both healthcare professionals and patients, creates a pressing need to understand the current state of IT adoption and utilization in Romanian hospitals [10]. While the country has improved its performance regarding the availability of e-Health data and continues to invest heavily in public service digitalization, the European Commission’s 2025 Digital Decade Country Report recommends that Romania continue expanding online health data sources and adopt a comprehensive strategy for health system digitalization that prioritizes user needs and ease of use. However, broader digital challenges persist, including underperformance in basic digital skills and difficulties retaining IT talent, which directly impact digitalization initiatives [11]. These ongoing efforts, combined with the need for user-focused implementation strategies, create an urgent need to understand how Romanian hospitals currently utilize IT systems and what barriers exist to more effective digital adoption. Romania’s EHR implementation remains constrained by systemic healthcare fragmentation, including incomplete provider data integration, insufficient training in data management and analysis, diverse incompatible software systems across hospitals, limited national health registries, and inadequate funding and personnel resources. Moreover, the digitalization of the Romanian health sector is hampered by a health information system that lacks an integrated, patient-centered approach and an effective process of data collection and validation. Consequently, public health policies often lack robust evidence and reliable impact assessments. These structural challenges prevent Romania from fully utilizing EHR capabilities. To address these limitations, the National Health Insurance House has been implementing the national EHR (called DES) project with European funding, which aims to establish comprehensive connectivity between EHR systems and all healthcare and health technology providers [12], but no updates have been provided over the last years.

The concepts designating electronic records in healthcare remain inconsistently defined [13]. Generally, electronic medical records (EMRs) are systems confined to a single organization, designed to replace paper-based files, whereas Electronic Health Records (EHRs) are broader, interoperable solutions that integrate longitudinal patient data from multiple sources [13,14,15,16]. However, the literature shows that this distinction is often blurred, as modern EMRs increasingly incorporate EHR-like functionalities [17]. In this study, we maintain this differentiation to emphasize the importance of interoperability and to clarify situations where patient data are, or are not, fully integrated into an electronic record.

Despite sustained policy commitment and ongoing investment, structural and skills-related barriers continue to slow the digitalization of healthcare in Romania; fully realizing the potential of the EHR and other digital solutions requires a coherent, user-centered strategy coupled with genuine, system-wide interoperability. Within this framework, turning digital gains into effective and equitable access makes it essential to complement digital transformation with service-planning and organizational policies. In addition to digitalization, a strategic approach to the allocation of physical and human resources is also required, in line with international recommendations on the spatial optimization of healthcare services [18]. The findings of Chen et al. (2023) [19] further emphasize that spatial access to care is shaped not only by medical infrastructure but also by population distribution and transportation networks. In areas with poor infrastructure, optimizing the location of populations and medical resources has an immediate impact, while improvements in transportation generate an even stronger marginal effect. Together, these insights highlight that digital transformation must be accompanied by systemic planning of healthcare resources and accessibility, particularly relevant for the Romanian context. Therefore, this study addresses a critical knowledge gap by examining how healthcare institutions are currently leveraging digital technologies and identifying barriers to more comprehensive digitalization, providing essential insights for policy development and strategic planning in Romania’s healthcare digital transformation.

A defining feature of the digital era in healthcare is the shift toward consumer-centric models, empowering patients to take greater responsibility for their health and participate actively in decision-making [3,6]. Digital health technologies facilitate patient empowerment, self-management, and shared decision-making, while enabling more personalized, goal-oriented care [1,2]. The proliferation of online platforms, mobile apps, and telemedicine services allows patients to access medical information and care remotely, fostering a transition from episodic, facility-based care to continuous, connected, and remote healthcare [3,7].

Technological innovations such as telemedicine, wearable devices, AI, and big data analytics are at the forefront of this transformation. Telemedicine, for example, enhances access to care in remote and underserved areas, reduces costs, and improves diagnostic quality, particularly for chronic and elderly patients [4,6]. Wearable devices and Internet of Things (IoT) technologies enable real-time health monitoring, supporting preventive strategies and early interventions [3]. AI applications in diagnostics, treatment planning, and predictive analytics are making healthcare more efficient and personalized, while big data analysis supports more accurate and timely clinical decisions [2,4].

The integration of digital technologies also brings organizational and workforce implications. The emergence of new digital health professions and the need for existing professionals to acquire new competencies necessitate comprehensive education and training strategies [1,5]. While digital solutions can alleviate administrative burdens and optimize resource allocation, they may also introduce new strains on healthcare workers, particularly during the initial phases of adoption. The acceptability of digital technologies among both professionals and patients is a critical determinant of successful implementation and sustained use [1,20]. Despite the many advantages of digital health, several challenges and barriers persist. Issues such as digital literacy, access to technology, affordability, and online accessibility can exacerbate health inequities, particularly among socio-economically disadvantaged groups [21]. Digital transformation also raises concerns about data privacy and the protection of sensitive medical information, with cyberattacks posing significant threats to the safe and uninterrupted functioning of health systems [2,3,6]. Cultural and organizational resistance to digital innovation is another significant barrier, affecting healthcare providers, organizations, and patients alike. Factors such as usability, perceived threat, tradition, and self-efficacy influence the adoption and sustained use of digital health solutions [20]. Furthermore, the lack of interoperability and standardization across digital platforms can hinder the seamless exchange of health information and limit the potential benefits of digitalization [1,5].

Romania’s digital healthcare transformation has been supported by progressive legislative developments that established comprehensive legal frameworks for telemedicine and digital health services. The foundational legislative milestone occurred in 2018 when Government Emergency Ordinance No. 8/2018 formally incorporated telemedicine into Romanian law by amending the Health Reform of 2006, primarily addressing chronic medical personnel shortages in remote areas and promoting equitable healthcare access. The COVID-19 pandemic in 2020 accelerated regulatory development, and subsequent ordinances established emergency telemedicine frameworks. By 2022, Government Decision 1133/2022 had created comprehensive implementation norms, leading to a mature regulatory environment that supports the full spectrum of digital healthcare delivery [22]. As part of the EU, Romania adheres to General Data Protection Regulation (GDPR), but implementation in public healthcare faced persistent difficulties, including challenges with data anonymization, emergency access protocols, and maintaining the balance between privacy protection and effective care delivery. For instance, notable security breaches occurred as recently as 2024, when ransomware compromised 20 hospital databases, disrupting medical service reporting and state reimbursement processes for several days—an incident that proper compliance with GDPR could have prevented. Successful compliance in Romanian healthcare demands thorough understanding of regulatory requirements, particularly given the highly sensitive nature of health-related data processing [23]. Interestingly, Romania established groundbreaking legislation in 2023 by becoming the first nation worldwide to legally recognize citizens’ right to personalized medicine, responding to the EU Council’s 2015 personalized medicine policy framework. It grants patients legal entitlement to individualized healthcare based on their unique genetic and phenotypic characteristics, incorporating robust safeguards by requiring informed consent for each personalized medical intervention and mandating strict data protection compliance with GDPR standards [24].

Therefore, the digital transformation of healthcare is a multifaceted and ongoing process, driven by technological advancements, policy initiatives, and changing societal expectations. It holds the potential to enhance the efficiency, quality, and accessibility of health services, empower patients, and foster innovation across the sector. However, realizing these benefits requires addressing challenges related to equity, education, privacy, security, and stakeholder engagement, as well as fostering a culture of acceptance and adaptability within healthcare systems. In this regard, maximizing the benefits of digitalization requires not only institutional readiness but also adequate patient health literacy. In Romania, limited levels of health literacy hinder the effective use of digital tools and reduce the extent to which their potential advantages are perceived.

The objective of our study is to investigate the extent to which digitalization in Romania addresses the current needs of patients, employing a convergent analysis of both user perceptions and managerial perspectives.

**Conceptual framework**

The conceptual basis of this evaluation is grounded in Donabedian’s classic model (2005) [25], which structures healthcare quality into three interdependent dimensions: structure, process, and outcomes. In the context of digital transformation, *structure* is defined by technological infrastructure, information systems, interoperability, and data security policies; *process* refers to the effective use of digital technologies in service delivery (e.g., telemedicine, electronic health records, artificial intelligence); and *outcomes* include clinical performance indicators, operational efficiency, patient satisfaction, and equity of access. While Donabedian’s model provides a clinical and organizational perspective, the World Health Organization’s Health System Performance Framework [26] adds a macro-systemic dimension, linking system functions (governance, resources, service delivery) with key objectives such as quality, access, efficiency, equity, and sustainability. This complementarity enables an integrated evaluation of digital transformation at both the institutional and systemic levels. In addition, the assessment is contextualized with reference to the **E-Government Development Index (EGDI)**, which measures national digital capacity based on infrastructure, online services, and human capital [27], and the **Digital Decade eHealth Indicator**, which monitors the progress of EU member states in areas such as equitable access to electronic health records, cross-border data exchange, and interoperability [28]. These instruments provide an international comparative framework, allowing the study’s findings to be related to global trends and strategic objectives in healthcare digitalization. Thus, the adopted conceptual framework enables the analysis of how digital transformation influences healthcare quality and patient satisfaction, while situating the results within internationally recognized standards and objectives.

**Hypotheses derivation**

The analysis of specialized literature highlights the significant impact of digital technologies on the quality, safety, and efficiency of healthcare services. According to Donabedian’s model (2005) [25], structural improvements (digital infrastructure, interoperability) and process optimization (electronic health records, telemedicine, artificial intelligence) lead to superior outcomes for both patients and organizations, a relationship supported by recent empirical evidence [1,5,29,30,31]. On this basis, H1 was formulated: The implementation of digital technologies significantly contributes to the improvement of the quality of medical services and operational efficiency.

Furthermore, studies summarized in the literature show that digitalization supports the transition toward a patient-centered model of care by expanding access to services and improving communication with healthcare professionals through telemedicine, online platforms, and mobile applications [2,3,4,6,7]. These technologies enhance continuity of care, especially for patients in remote areas or with reduced mobility, and increase their satisfaction and engagement in the therapeutic process. On this basis, H2 was formulated: Digitalization has a positive impact on patient satisfaction, facilitating access to care and improving communication with medical staff.

## 2. Materials and Methods

The research adopted a cross-sectional, descriptive approach, incorporating two complementary tools: a mixed-methods questionnaire applied to patients and a qualitative questionnaire addressed to hospital managers, to enable data triangulation, following the convergent model recommended by Creswell & Creswell (2017) [32].

**The mixed-methods questionnaire for patients**

The patient questionnaire was conducted online between 6 and 14 May 2025 and included 21 questions (5 demographic and 16 on perceptions of medical digitalization). We employed a convenience sampling approach, distributing the online questionnaire through various social media channels to achieve broad demographic representation across different patient populations. This non-probabilistic sampling method was chosen to maximize accessibility and capture diverse perspectives from patients with varying healthcare experiences, ages, and backgrounds. This sampling strategy inevitably excluded patients without access to social media or the internet. However, since the study specifically focused on the perceptions of digital service users, this limitation is consistent with the research objectives and does not undermine the validity of the findings. Inclusion criteria for patients were as follows: age ≥ 18 years, having accessed healthcare services within the past 12 months, provision of informed consent, and the ability to independently complete the online questionnaire. Exclusion criteria included the inability to complete the questionnaire autonomously and lack of internet access.

The questions were adapted from validated instruments in the specialized literature (DHASSP, pSUAPP, HEALTHQUAL), covering aspects such as satisfaction, ease of use, impact on quality, and perceived barriers [33,34,35]. Additionally, to reflect aspects indirectly linked to digital literacy, such as familiarity with digital services, perceived benefits, usability challenges, or trust in technology, relevant foundational works on digital health competencies were referenced [36,37,38]. The mixed structure of the questionnaire, combining closed items with open-ended questions, was inspired by the pSUAPP scale model, which integrates quantitative and qualitative components to better capture the user experience [35]. These open-ended questions allowed for capturing subjective perceptions, barriers, and spontaneous suggestions from respondents. The questionnaire included: closed-ended questions with ordinal scales, including Likert-type scales (1–5); nominal single-choice questions; multiple-response questions (check-all-that-apply); and open-ended questions for an in-depth exploration of opinions. The open-ended questions allowed for capturing subjective perceptions, barriers, and respondents’ spontaneous suggestions. The questions and reply options are included in the Supplementary Material (Supplementary File S1).

The responses were recoded into ordinal categories, after which contingency tables were constructed to compare the observed frequencies with those expected under the assumption of independence. The conditions for application were checked (expected frequencies > 5), and the effect size was estimated using Cramér’s V coefficient, interpreted according to conventional thresholds (0.1 = weak, 0.3 = moderate, 0.5 = strong) [39]. The same methodological procedure was also applied to explore the relationships between demographic variables and patterns of technology use, wherever this was relevant for the interpretation of the results.

Results were considered relevant at 95% confidence level (*p* < 0.05).

Ordinal regression with a logit link function was applied to the ordinal dependent variables: perception of service quality improvement and patient satisfaction level, following methodological recommendations from the literature [40,41]. Predictors included binary variables related to the use of each type of digital service (e.g., electronic prescription, online appointment, mobile applications, virtual consultations), perceptions of the benefits of digitalization (e.g., easier access, risk reduction, improved communication), as well as control variables to reduce confounding effects: age, gender, education level, place of residence, and frequency of digital service use [42].

Data were analyzed using Microsoft Excel 2021 and IBM SPSS Statistics 29, employing descriptive statistics, chi-square tests, and ordinal regression, with models validated by standard coefficients (Nagelkerke R^2^, Cramér’s V, OR). In addition, multiple-choice items were converted into indicator variables and, when needed, restructured into tuples (e.g., service × satisfaction) to allow contingency testing. Associations were first examined with chi-square, supplemented by Cramér’s V as an effect size less sensitive to sample size, and by Kendall’s τb for ordinal variables. For multivariate analysis, we estimated ordinal logistic regressions (proportional odds model, logit link), explicitly testing the proportional odds assumption. Full regression results are reported, including coefficients, odds ratios, 95% confidence intervals, and *p*-values, to ensure transparency. Given the number of predictors, Bonferroni corrections were applied to reduce the risk of type I error.

**The qualitative questionnaire for hospital managers**

For the institutional perspective, data collection was conducted through the Administration of Hospitals and Medical Services of Bucharest, which acted as an intermediary to distribute and centralize responses. A qualitative questionnaire with open-ended questions was sent to 25 public hospitals in Bucharest, during the period 3–24 March 2025. While smaller than the patient sample, this number falls within the range commonly reported in qualitative health research, where 10–20 participants are often considered sufficient to achieve thematic saturation. As highlighted by Vasileiou, Barnett, Thorpe, and Young (2018) [43], the adequacy of a qualitative sample is determined less by statistical representativeness and more by the study’s objectives, the specificity of the context, and the attainment of theoretical saturation. In this study, the selection of managers reflects the aim of generating in-depth insights into institutional practices and barriers, rather than pursuing statistical generalization, and is therefore consistent with established international standards in qualitative health research. The inclusion criterion for managers was to be holding an administrative or decision-making position in the hospital during the study period; exclusion criteria were the absence of an active administrative role or refusal to participate. The questionnaire addressed to managers was developed based on the digital maturity assessment frameworks issued by the World Health Organization (2023) [44] and the Organisation for Economic Co-operation and Development (2023) [45], and was further contextualized with reference to the E-Government Development Index (United Nations, 2024) [27] and the Digital Decade eHealth Indicator (European Commission, 2025) [28]. The domains covered included: digital infrastructure, interoperability, telemedicine, patient access to data, ongoing projects, and organizational barriers. The questionnaire was composed exclusively of open-ended questions, without a standardized response format, in order to allow for the free expression of managerial perspectives. No personal or institutional identifying information about individual managers or hospitals was collected. The questionnaire is included in the Supplementary Material (Supplementary File S2). Responses were analyzed through thematic coding and, where possible, interpreted quantitatively for triangulation.

**Data triangulation**

Data triangulation was carried out using a convergent parallel design, in which quantitative datasets (patient questionnaire) and qualitative datasets (open-ended questionnaire for managers) were analyzed separately, employing methods specific to each (descriptive and inferential statistical analyses for quantitative data, and deductive–inductive thematic coding for qualitative data, using the WHO and OECD digital maturity frameworks as starting points) [44,45].

The results were then mapped onto corresponding themes, which enabled the identification of both convergence points (e.g., patient priorities for telemedicine and the electronic health record, confirmed by national strategic directions) and discrepancies. These divergences were further examined to identify explanatory factors such as patients’ access to services outside the studied institutions, lack of interoperability, low digital literacy, or differences in the definition of digital services.

This mixed-methods approach allowed for correlating user perceptions with institutional realities reported by managers, thereby strengthening the validity of conclusions and the relevance of the formulated recommendations, while being interpreted through Donabedian’s structure–process–outcome model to ensure consistency with the conceptual framework.

## 3. Results

### 3.1. Sample Description of the Quantitative Analysis—Patient Questionnaire

**Figure 1:**
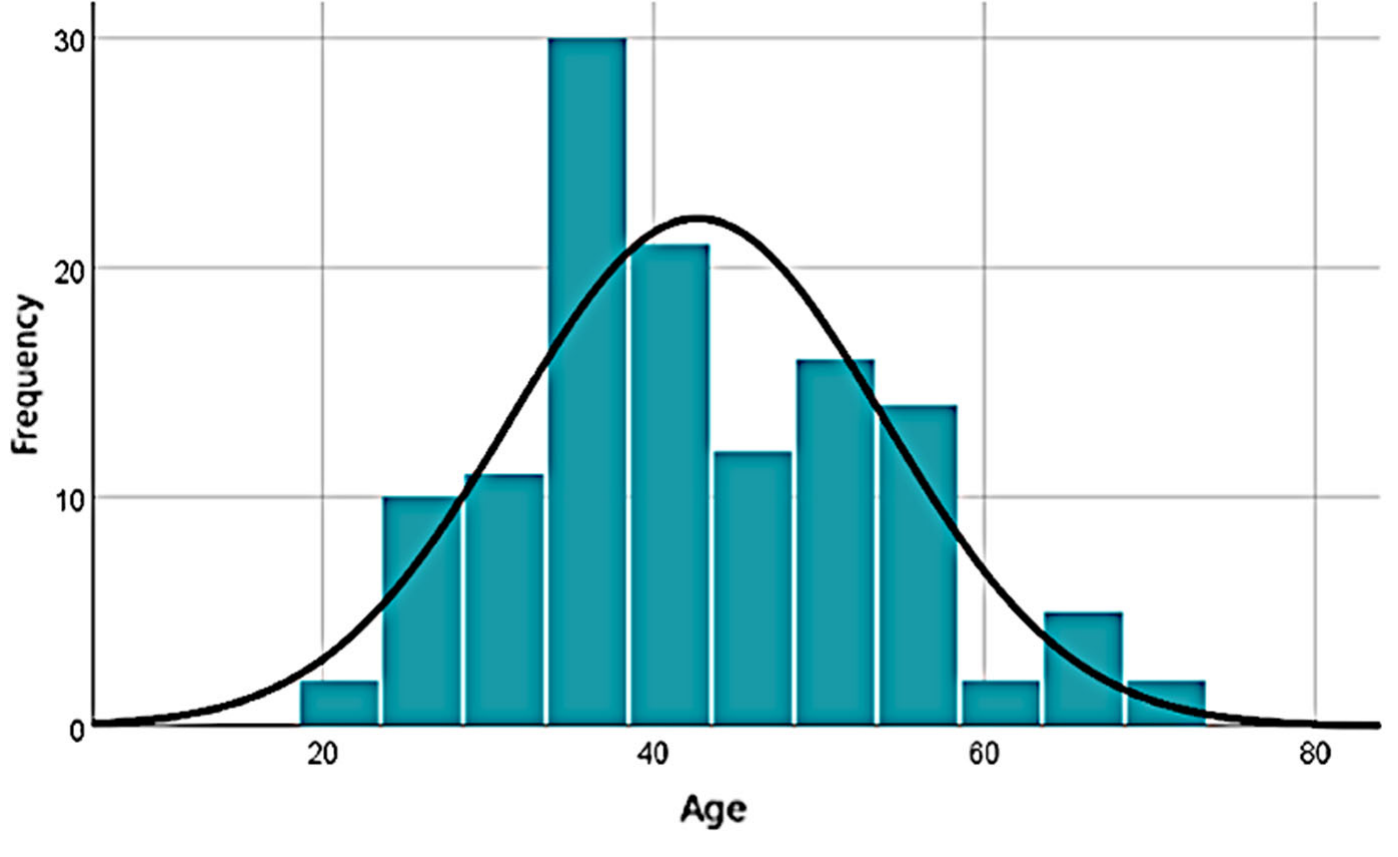
Distribution of Patients by Age. Source: Authors, based on analysis performed in IBM SPSS Statistics (version 29) and Microsoft Excel.

The analyzed sample consists predominantly of active adult individuals, with respondents’ ages ranging from 21 to 73 years, a mean age of 42.66 years, and a median of 40, indicating a slightly positive skew in distribution. The standard deviation of 11.26 years reflects moderate dispersion, and the absence of missing data enhances the robustness of the analysis. The wide range (52 years) highlights significant demographic diversity within the sample, supporting the relevance of this variable in subsequent statistical analyses (Figure 1). The majority of respondents are female (72.8%), from urban areas (85.6%), and hold higher education degrees (63%).

### 3.2. Use of Digital Medical Services

**Figure 2:**
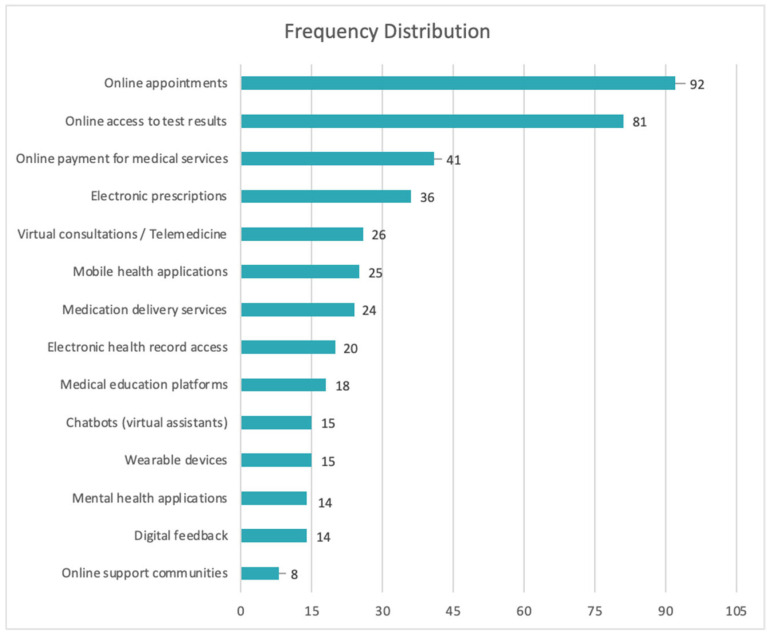
Frequency Distribution of the Types of Digital Medical Services Used by Respondents. Source: Authors, based on analysis performed in IBM SPSS Statistics (version 29) and Microsoft Excel.

An analysis of the responses from the 125 patients reveals that 81.6% of respondents (*n* = 102) have used at least one digital medical service. The most frequently used functionalities are administrative in nature: online appointment scheduling (73.6%), access to medical test results (64.8%), digital payment for services (32.8%), and electronic prescriptions (28.8%). Advanced services, such as applications for health monitoring or psychological support, are significantly less utilized: only 11.2% used mental health apps, and 6.4% participated in online support communities (Figure 2).

Only 33.6% of respondents reported having used telemedicine to access services that were unavailable locally, while 48% did not use such services at all. Additionally, the level of active participation remains low: only 12.8% provided digital feedback on the medical services they used.

### 3.3. Patients’ Perception of the Impact of Digitalization in Healthcare

The collected data show that 65.6% of respondents believe that digitalization has improved access to medical services, while 28% expressed a neutral opinion. Approximately 50% of patients believe that digitalization has improved communication with doctors, mainly through the use of phones or messaging apps. Specialized digital platforms are less frequently used as the primary means of communication.

**Figure 3:**
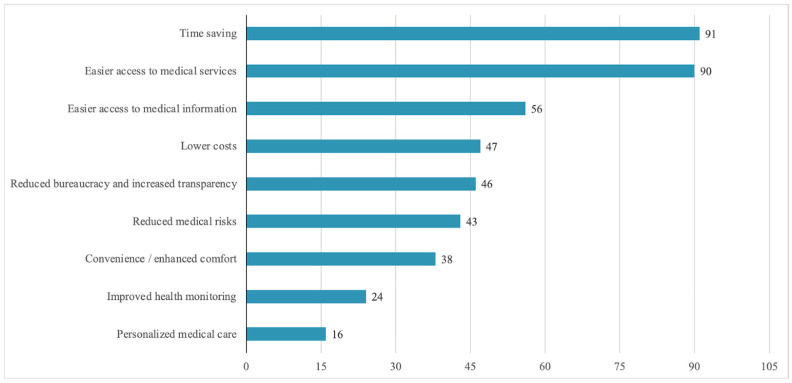
Ways in Which the Quality of Medical Services Has Been Improved Through Digitalization. Source: Authors, based on analysis performed in IBM SPSS Statistics (version 29) and Microsoft Excel.

The majority of respondents (94%) evaluate the impact of digitalization on service quality positively. However, when asked about specific ways in which quality has improved, they mainly highlight functional benefits (Figure 3): time savings (72.8%), easier access to services (72%), access to medical information (44.8%), and cost reduction (37.6%). Advanced clinical benefits are rarely mentioned: health monitoring (19.2%) and treatment personalization (12.8%).

Regarding overall satisfaction with digital services, **87.2%** of patients report a favorable impact, with **48%** expressing a high level of satisfaction, compared to **39.2%** who report moderate satisfaction.

### 3.4. Barriers, Risks, and Concerns Regarding Digitalization

**Figure 4:**
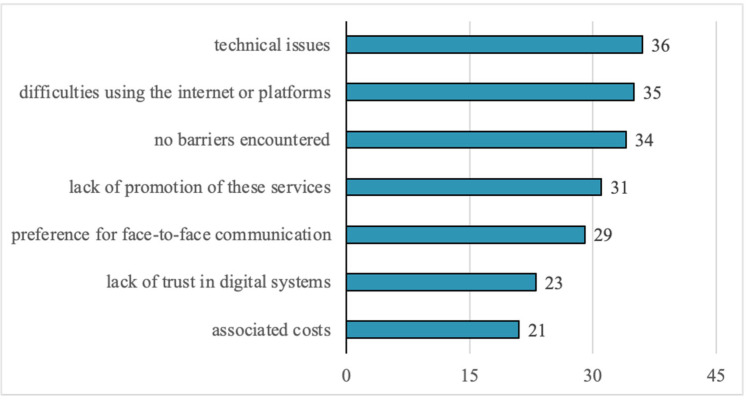
Main Barriers to Using Digital Medical Services. Source: Authors, based on analysis performed in IBM SPSS Statistics (version 29) and Microsoft Excel.

The main barriers reported by respondents in using digital medical services include: technical issues (28.8%), difficulties using the internet or digital platforms (28%), lack of service promotion (24.8%), a preference for face-to-face interactions (23.2%), and lack of trust in digital systems (18.4%). Only 16.8% of respondents reported no barriers (Figure 4).

**Figure 5:**
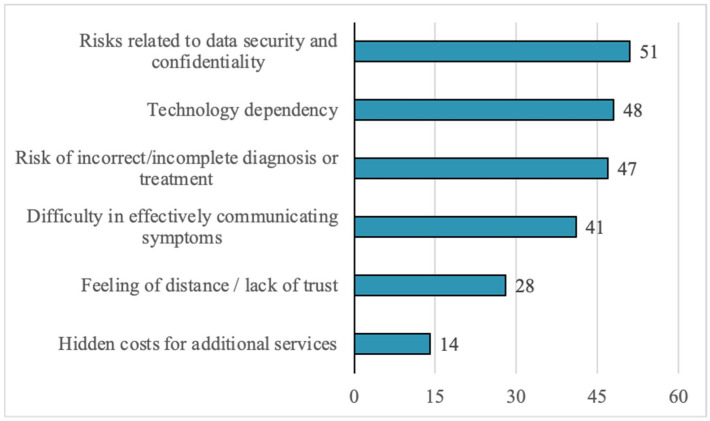
Main Risks Associated with Digital Medical Services. Source: Authors, based on analysis performed in IBM SPSS Statistics (version 29) and Microsoft Excel.

Regarding perceived risks, the most frequently mentioned include: data security and confidentiality (40.8%), dependence on technology (38.4%), and the risk of incorrect diagnosis or treatment (37.6%). Other cited risks include difficulties in communicating symptoms (32.8%) and a sense of detachment or impersonality (22.4%) (Figure 5).

**Figure 6:**
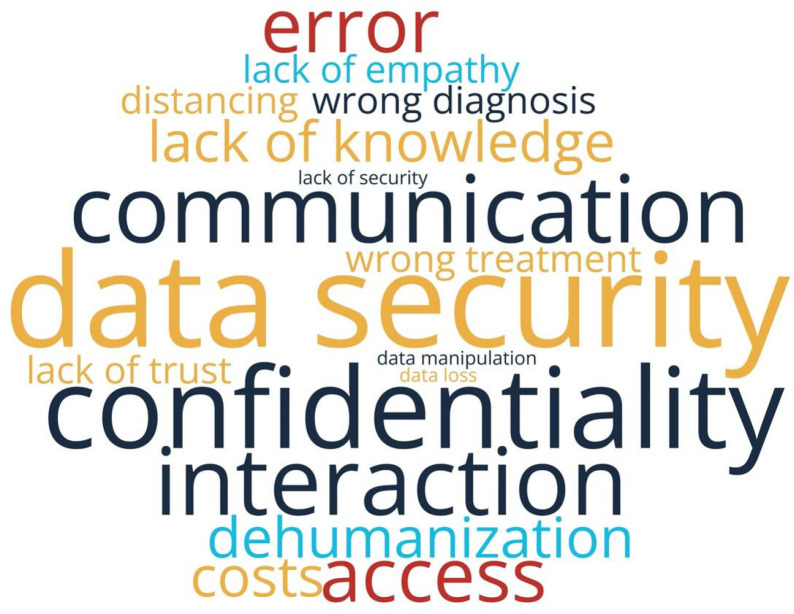
Word Cloud Showing the Frequency of Keywords Identified in Patients’ Responses About Concerns Related to Health Digitalization.

In response to the open-ended question about concerns related to digitalization, respondents frequently highlighted: risks to the confidentiality of medical data, the potential dehumanization of the doctor–patient relationship, and inequalities in access to technology. The answers reflect concerns about digital exclusion, especially among vulnerable individuals or those living in areas with limited infrastructure (Figure 6).

The perception of inequalities between the public and private sectors is pronounced among respondents: 75.2% believe there are significant differences in the level of digitalization between the public and private healthcare sectors. The private sector is perceived as being more advanced in adopting digital technologies, while the public system is associated with challenges related to infrastructure, resources, and implementation.

### 3.5. Attitudes Toward the Future of Health Digitalization

**Figure 7:**
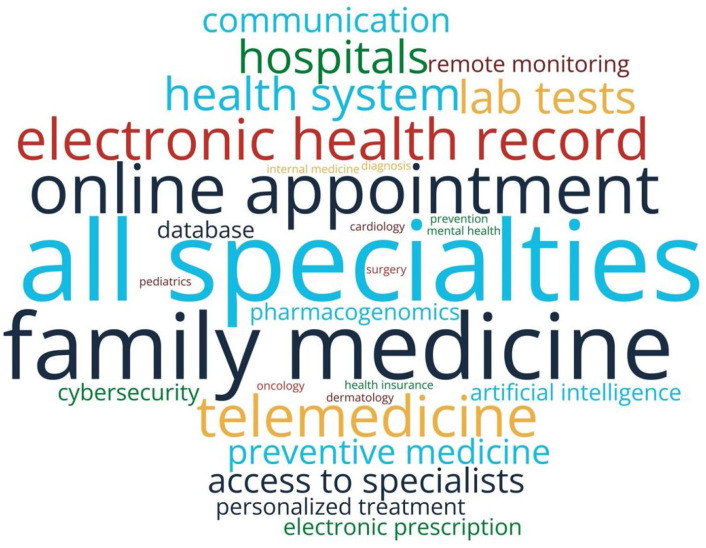
Word Cloud Showing the Frequency of Keywords Identified in Patients’ Responses Regarding their Perceived Priorities of Digitalization in Medicine.

The analysis of responses reveals a predominantly favorable attitude toward the future of digitalization in healthcare: 87.2% of respondents support the development of digital medical services in Romania, while 12.8% do not consider this initiative necessary. Among the top priorities identified by patients are: expansion of telemedicine services, development of the electronic health record, optimization of medical appointment systems, strengthening of family medicine, enhancement of cybersecurity, and integration of artificial intelligence (Figure 7).

### 3.6. Hypotheses Validation

#### 3.6.1. The Use of Digital Medical Services Used (Q7) and Perceived Quality of Care (Q8)

**Figure 8:**
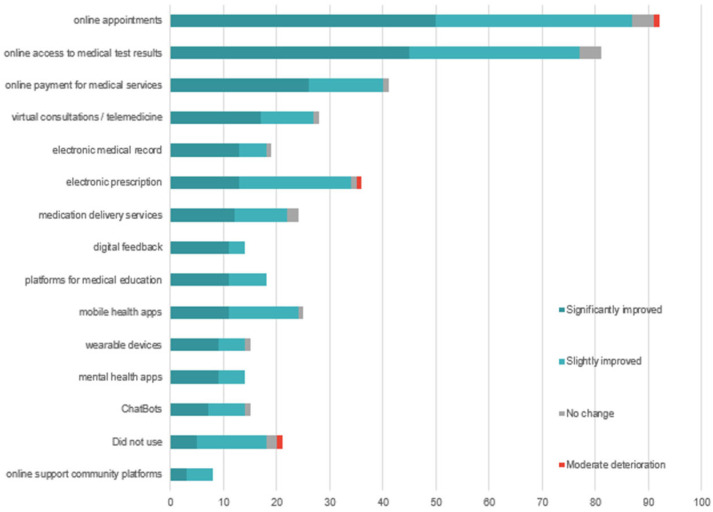
Perceived Impact of Digital Health Services on Quality of Care: Cross-tabulation by service type and satisfaction level. Source: Authors, based on analysis performed in IBM SPSS Statistics (version 29) and Microsoft Excel.

To test Hypothesis H1, which states that the implementation of digital technologies significantly contributes to the improvement of the quality of medical services (and operational efficiency), the relationship was analyzed between patients’ perception of service quality following digitalization (dependent variable—Question 8) and the actual use of various types of digital medical services (independent variable—Question 7), as illustrated in Figure 8.

To test the hypothesis that the use of digital medical technologies influences patients’ perception of healthcare quality, we restructured the survey data into a tall format in which each selected technology ever in use was represented as a separate record, and linked to the respondent’s perceived quality rating (Q8). In the new table, each participant contributes between one and fifteen records, depending on the number of technologies they reported using. This generated 465 valid service-perception pairs, which we summarized in a 15 × 4 contingency table (technology × perceived quality). We analyzed the tall table, initially, with the square test of independence. The Pearson chi-square test did not reveal a significant association between the two variables, with χ^2^(42, N = 456) = 37.32 corresponding to *p* = 0.676. The effect size, measured by Cramér’s V = 0.165, indicates a very weak relationship with no practical relevance. Even when treating perceived quality as an ordinal outcome, Kendall’s tau-b similarly failed to confirm a systematic trend across technology categories (τb = −0.041, *p* = 0.308).

#### 3.6.2. Ordinal Regression Model on Perceptions of Improved Quality of Medical Services Through Digitalization

To analyze how the perception of improved medical service quality through digitalization is influenced by the use of digital technologies and communication experiences with medical staff, a complementary ordinal regression model was developed.

To further explore the relationship, we estimated an ordinal logistic regression (logit link, proportional odds model) with perceived service quality (Q8, four ordered levels) as the dependent variable and the use of specific digital medical technologies, represented by the surrogate variables Q7a–Q7n, as predictors. We did not include the fifteenth surrogate variable corresponding to “non-use” in order to avoid collinearity. The overall model was statistically significant, χ^2^(14) = 34.13, *p* = 0.002, indicating that the inclusion of technology-use variables improved model fit relative to the null. Goodness-of-fit tests supported the adequacy of the proportional odds assumption (Pearson χ^2^ *p* = 0.998; Deviance *p* = 1.000). The explained variance was moderate (Nagelkerke R^2^ = 0.284).

At the predictor level, the ordinal logit coefficients translated into substantially different odds of reporting higher quality. Surprisingly, the strongest predictors, from a statistical significance point of view, are associated with a less favorable perception regarding the quality of the digital medical process (Table 1). Use of electronic prescriptions was associated with significantly lower odds of perceiving service quality as improved (OR = 0.23, 95% CI: 0.08–0.64, *p* = 0.005), as was the use of mobile health apps (OR = 0.23, 95% CI: 0.06–0.82, *p* = 0.024). Medication delivery showed a similar tendency, though only marginally significant (OR = 0.34, 95% CI: 0.11–1.0, *p* = 0.056). To account for multiple testing, we applied a Bonferroni correction across the 14 predictors, yielding a significance threshold of α = 0.05/14 = ~0.0036. Under this stringent criterion, none of the predictors reached statistical significance. The association between electronic prescriptions and lower odds of reporting improved service quality (unadjusted *p* = 0.005) came closest, but did not survive correction. Thus, while electronic prescriptions appear to be the strongest individual predictor, all results should be interpreted with caution in light of the conservative adjustment. The diversity of services is matched by the ample range of *p* values and ORs, indicating that patients interact in distinct, service-dependent manner with the digital medical technologies, thus achieving variable degrees of quality perception.

#### 3.6.3. The Impact of Medical Digitalization on Patient Satisfaction

To test Hypothesis H2, which states that digitalization has a positive impact on patient satisfaction by facilitating access to care and improving communication with medical staff, relationships were analyzed between reported satisfaction levels (dependent variable—Question 10) and two independent variables: types of digital medical services used (Question 7) and perceptions of specific benefits of digitalization (Question 9).

### 3.7. Types of Digital Medical Services Used and Patient Satisfaction (Q7 × Q10)

The purpose of the following analysis is to examine the relationship between the types of digital medical services used by respondents and their perception of the impact of digital technology adoption on satisfaction with medical services.

**Figure 9:**
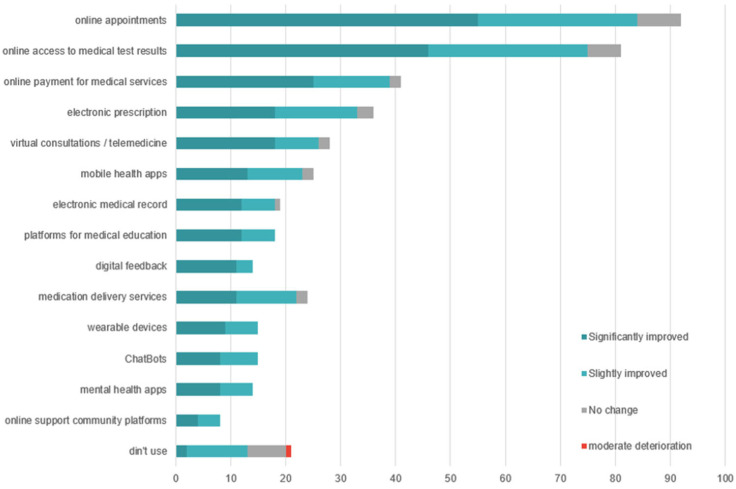
Cross-tabulation of the Types of Digital Health Services and Patient Satisfaction Levels (Q7 × Q10). Source: Authors, based on analysis performed in IBM SPSS Statistics (version 29) and Microsoft Excel.

The majority of respondents who used digital medical services such as online appointment scheduling (*n* = 92), access to online test results (*n* = 81), online payment (*n* = 41), and electronic prescriptions *(n* = 36) reported a significant or moderate improvement in satisfaction. For example, 55 of those who used online appointment scheduling and 46 of those who accessed investigation results online reported a significant improvement in satisfaction. In contrast, among respondents who did not use digital services (*n* = 21), 7 reported no change, and 1 reported a moderate decline in satisfaction (Figure 9).

The relationship between types of digital medical services and patient satisfaction (Q7 × Q10) was first assessed through chi-square analysis. Similar to the test for the H1 hypothesis, we constructed a secondary table comprising 456 valid cases where a technology was mentioned in tandem with a satisfaction report. This table was summarized in a 15 × 4 contingency table, which subsequently yielded a significant Pearson χ^2^ statistic, χ^2^(42) = 64.11, *p* = 0.016. This indicates that the distribution of satisfaction levels differed depending on the service used. However, the overall effect size was modest (Cramer’s V = 0.216). Moreover, Kendall’s tau-b (τb = −0.064, *p* = 0.109) did not support a consistent monotonic trend across the ordinal structure of satisfaction responses.

Subsequently, we estimated the same monotonic trend with an ordinal logistic regression model (logit link, proportional odds model), using satisfaction level (Q10) as the dependent variable and, as predictors, the same fourteen surrogate variables (Q7a–Q7n, excluding “did not use”), describing the use of specific services, as before. The model was statistically significant, χ^2^(14) = 35.20, *p* = 0.001, and showed good fit to the data (Pearson *p* = 1.000; Deviance *p* = 1.000). Pseudo-R^2^ values suggested a moderate share of explained variance (Nagelkerke R^2^ = 0.283).

At the individual technology level, several services were associated with substantial differences in odds of reporting higher satisfaction (Table 2). Online appointment scheduling emerged as the strongest positive predictor (OR = 5.1, 95% CI: 1.9–13, *p* = 0.001), followed by medication delivery (OR = 0.25, 95% CI: 0.09–0.74, *p* = 0.013), the latter predicting, surprisingly, a decline in satisfaction ratings among users, compared to non-users. In addition, platforms for medical education showed a positive tendency (OR = 3.6, 95% CI: 0.92–14, *p* = 0.065), though this did not reach conventional significance. Applying a Bonferoni correction for multiple comparisons reduced the number of proven predictors to only one, namely the use of online appointment scheduling. Overall, the results suggest that not all digital tools are equally valued by patients.

### 3.8. Perceived Benefits of Digitalization and Patient Satisfaction (Q9 × Q10)

**Figure 10:**
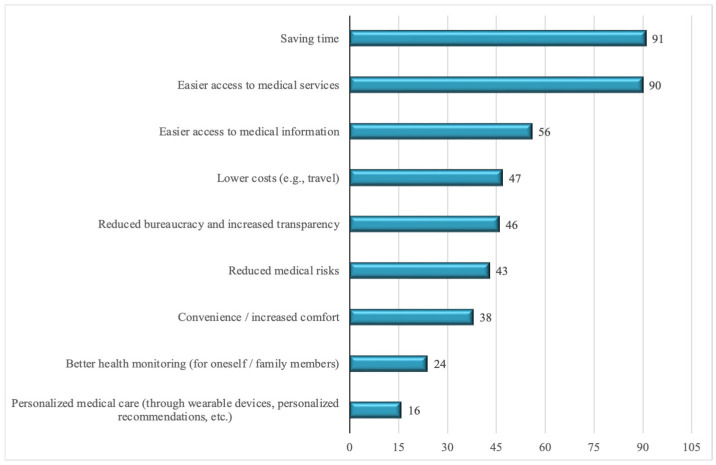
Perceived Benefits of Digital Health Services and Their Association with Patient Satisfaction: Cross-tabulation by Perceived Benefits (Q9) and Satisfaction Level (Q10). Source: Authors, based on analysis performed in IBM SPSS Statistics (version 29) and Microsoft Excel.

The analysis of the relationship between the perceived benefits of digitalization (Question 9) and patient satisfaction (Question 10) reveals a clear trend: respondents who mentioned easy access to services (*n* = 90), time savings (*n* = 91), and reduced medical risks (*n* = 43) frequently reported a high level of satisfaction. For example, 53 of those who identified easy access and 47 of those who mentioned time savings reported a significant improvement in satisfaction. In contrast, respondents who were unable to identify a specific benefit (*n* = 7) mostly reported no change or even a decline in satisfaction (Figure 10).

The relationship between perceived benefits of digitalization (Q9) and patient satisfaction (Q10) was first examined using a contingency framework. We created a secondary table comprising all the cases when a respondent stated a perceived benefit of digitalization in conjunction with a general satisfaction score. The 458 such cases were counted in a contingency table with 9 perceived benefits × 4 levels of satisfaction. This latter table corresponds to a highly significant Pearson χ^2^ statistic, χ^2^(27) = 126.23, *p* < 0.001, indicating that satisfaction levels differed depending on the specific benefits perceived. The overall association strength was moderate (Cramer’s V = 0.303). However, Kendall’s tau-b (τb = −0.031, *p* = 0.447) did not support the existence of a consistent monotonic trend across satisfaction levels.

To account for the ordinal structure of Q10, we next estimated an ordinal logistic regression model (logit link, proportional odds), with satisfaction as the dependent variable and the nine benefit indicator variables (Q9a – Q9i), excluding the collinear indicator of complete benefit absence, as predictors. The model was statistically significant, χ^2^(9) = 32.02, *p* < 0.001, and goodness-of-fit measures indicated an excellent fit (Pearson *p* = 0.988; Deviance *p* = 0.999). Pseudo-R^2^ values suggested a moderate explanatory power (Nagelkerke R^2^ = 0.260).

At the predictor level (Table 3), easier access to medical services was the only benefit strongly associated with higher odds of reporting improved satisfaction (OR = 5.1, 95% CI: 2.1–12.4, *p* < 0.001). None of the other benefits showed a statistically significant effect at the conventional threshold. In contrast, even after applying a Bonferroni correction for multiple testing, “easier access to medical services” retained statistical significance. This highlights that, while patients mention several potential advantages of digitalization, satisfaction is most strongly and consistently tied to improved access to care.

Therefore, patient satisfaction is influenced not only by the use of digital services but also by a clear perception of the added value that digitalization brings to the medical act.

### 3.9. Interpretation of the Ordinal Regression Model Regarding Patient Satisfaction

To assess the influence of using digital medical services on the level of perceived patient satisfaction, an ordinal regression model with a logit link function was applied. The dependent variable was the satisfaction reported following digitalization, while the predictors included variables describing the use of various digital medical technologies (e.g., online appointments, mobile apps, virtual consultations, etc.), perceptions of communication, platforms used, and accessed digital functionalities.

The final test proposed for the Hypothesis H2 examined whether patient satisfaction with digitalization (Q10) can be predicted jointly by the use of specific digital medical technologies (Q7), the perceived benefits of digitalization (Q9), the platforms employed for digital communication with physicians (Q12), and the level of communication with providers (Q11). To this end, an ordinal logistic regression with a logit link and proportional odds was estimated, with Q10 as the outcome and all aforementioned items as predictors. In preparation for this step, the item Q12 was replaced by five surrogate items indicating the use of each specific communication method (telephone, email, WhatsApp, etc.). The overall model was highly significant, χ^2^(29) = 72.32, *p* < 0.001, with excellent fit indices (Pearson *p* = 0.967; Deviance *p* = 1.000). The explanatory power was notable, with Nagelkerke R^2^ = 0.506, indicating that around half of the variability in satisfaction could be explained by the included predictors.

At the individual predictor level (Table 4), again, easier access to medical services was by far the strongest positive predictor (OR = 8.17, 95% CI: 2.52–26.5, *p* < 0.001), substantially increasing the odds of reporting higher satisfaction among those endorsing this benefit compared to others. Online appointment scheduling also predicted higher satisfaction (OR = 4.36, 95% CI: 1.28–14.8, *p* = 0.019). Conversely, medication delivery was associated with markedly lower satisfaction (OR = 0.11, 95% CI: 0.03–0.41, *p* = 0.001), suggesting that expectations may not align with perceived service outcomes in this area. In addition, perceiving less bureaucracy and more transparency showed a modest but significant effect (OR = 0.26, 95% CI: 0.07–0.89, *p* = 0.033), unexpectedly in the negative direction.

Other predictors, including the choice of digital communication platforms (Q12) and reported changes in communication with providers (Q11), did not reach conventional levels of significance. After correcting for multiple comparisons (Bonferroni adjustment), only “easier access to medical services” retained robust statistical support.

Overall, the results suggest that while many facets of digitalization are appreciated, the strongest determinant of patient satisfaction remains the perception that digital technologies facilitate access to care. This underscores the centrality of accessibility as a driver of satisfaction in digital healthcare transformation.

We also employed an adjusted model, which controls for age, gender, education level, urban setting, and occupation, variables that are commonly used in controlling for bias [42]. The adjusted model exhibited the same patterns as the unadjusted model, with the concern for ease of access to medical services being the only predictor that showed statistical significance, even after Bonferroni correction.

### 3.10. The Qualitative Questionnaire for Hospital Managers

The data obtained from the managers’ responses (N = 15) reflect that hospital digitalization processes are focused mainly on internal information management, lacking patient-oriented functionalities and showing a low level of implementation of advanced digital solutions. All 15 hospitals use IT systems exclusively for internal purposes, primarily for reporting, archiving, and appointment scheduling, without providing patients access to their own medical records or interactive digital platforms. Patient access to their own medical data is nonexistent in all 15 hospitals. No manager reported the implementation of a platform that allows patients to consult their digital medical files or access paraclinical results, highlighting a lack of transparency and active patient involvement. This finding is confirmed by managerial testimonies: the representative of Hospital 2 underlined that the system “is used internally, only within the medical unit that implements it (…), and access to patient data is restricted exclusively to our institution,” while the manager of Hospital 8 emphasized that “there is no integrated system that facilitates interoperability between different hospitals; the transfer of patients is carried out directly by the attending physician.” By contrast, Hospital 12 expressed a more optimistic perspective, noting that “patients’ data are automatically collected and entered into the electronic record (…), and accessing data from hospitalizations in other units is currently non-functional, but there is confidence that the system will soon become operational.” These examples illustrate that the problem is not only institutional, but systemic, linked to the lack of national interoperability.

Telemedicine services are used only marginally in the analyzed hospitals. Only 13.3% of units reported regular use of such solutions, while 26.7% mentioned occasional initiatives without a broad scope or systemic integration. The examples provided include usage limited to certain clinical departments, without a tele-assistance component, partial application in current activities, periodic interdisciplinary meetings, or individual, limited access of medical staff to digital tools. These findings indicate uneven implementation, restricted applicability, and no grounding in a clear institutional strategy for integrating telemedicine. The majority of facilities (60%) do not use telemedicine services, indicating the absence of a coherent strategy in this area. This situation is also reflected in managers’ statements: most hospitals declare telemedicine absent (Hospitals 1, 2, 4, 5, 9, 10, 13, 14, 15) or minimally used (Hospitals 3, 11, 12). Hospital 7 highlighted that “a telemedicine system was implemented during the pandemic, which is still functional today,” while Hospital 8 uses telemedicine and tele-assistance “in various situations, such as consultations and monitoring, when patients need these services without having to come to the hospital.” Nevertheless, usage remains very low; for example, Hospital 11 reports a 7% adoption rate, while in some cases it is reduced to interdisciplinary collaboration, as in Hospital 12, where the Oncology Commission meets biweekly, a practice closer to tele-assistance than to actual telemedicine.

Out of the 15 hospitals analyzed, only 7 units (46.7%) reported having ongoing projects aimed at digitalizing medical services. The remaining 8 hospitals (53.3%) stated that they are not currently undertaking any such initiatives. This distribution highlights uneven institutional development, with significant differences in hospitals’ capacity to initiate and support digital transformation processes. The contrast is evident between Hospitals 4 and 14, where digitalization remains minimal and IT systems are used almost exclusively for administrative functions (patients, medications, analyses, accounting, HR), but managers reported no major difficulties with the current processes. By contrast, Hospital 5, which relies on the same platform, highlighted multiple limitations and barriers: *“deficient infrastructure, insufficient staff training, limited accessibility, difficulties with SIUI (Integrated Single Information System), and cybersecurity vulnerabilities.”* This divergence illustrates that digital maturity depends not only on the technical infrastructure in place, but also on how the system is perceived, experienced, and managed at the institutional level.

Regarding interoperability, 93.3% of hospitals (14 out of 15) do not have a functional system for exchanging information with other medical institutions. The only exception is one hospital that partially uses a system allowing the transfer of imaging investigation only, without integrating other categories of clinical patient data.

**Figure 11:**
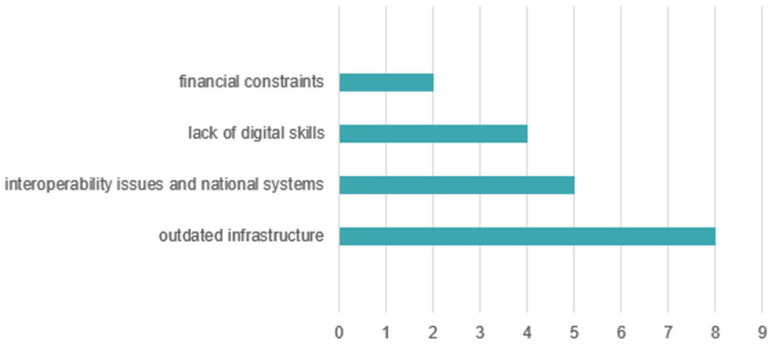
Challenges Identified by Managers Across the 15 Analyzed Hospitals. Source: Authors, based on analysis performed in IBM SPSS Statistics (version 29) and Microsoft Excel.

The challenges in digitalizing the units, from the perspective of the 15 managers participating in the study, can be grouped into four main categories of challenges that reflect the difficulty in implementing digital systems (Figure 11), noting that some managers identified one or more challenges.

The first theme is outdated infrastructure, explicitly mentioned in 8 of the 15 responses. This issue is perceived as a major obstacle to the effective implementation of digitalization. Within this theme, terms such as “IT equipment,” “high-performance servers,” or “mobile devices” represent the challenges faced by managers, and in some cases, the difficulties of optimally integrating these components into existing systems are also emphasized. The manager of Hospital 3 detailed: “Infrastructure (network/cabling)—very old, approx. 26 years (…). Terminals—theoretically updated, but upgrades depend on the budget (…). External—problems with National Health Insurance House (CNAS) servers (…). The human factor—the awareness of healthcare staff and patients regarding digitalization is very important.” These remarks show that digitalization is shaped not only by technical and financial limitations but also by users’ readiness and involvement. Nevertheless, the same manager added, “At present, in our hospital, there are no ongoing projects aimed at investing in technology.”

The second theme is the lack of digital competencies among staff, mentioned in four responses. Managers report difficulties in adapting medical personnel to new technologies, a lack of training, and general reluctance to use digital systems.

Issues related to interoperability and national systems represent the third theme, brought up by 5 managers. These responses highlight challenges related to the lack of interoperability between systems, particularly in connection with national applications such as SIUI, with frequent mentions of “discontinuous connection” and “application limitations.” The managers of Hospital 7 pointed out that “most of the problems we face come from the national SIUI, Prescriptions, and SIUI-Insured applications, which are managed by CNAS.” Hospital 9 emphasized similar barriers: “the main challenges remain the lack of connection with management systems in other hospitals, difficult accessibility to the DES system, discontinuous connection with the CAS server, and difficulties in issuing reimbursed prescriptions.” Likewise, Hospital 13 reported that “the greatest challenges are related to uploading both medical and financial data into SIUI.” These testimonies underline that many digitalization barriers cannot be solved exclusively at the institutional level, but require coordinated national solutions.

The final theme relates to financial constraints, present in two responses, where lack of funding is mentioned as a barrier both for acquiring infrastructure and for maintaining and expanding existing systems. Additionally, four managers did not indicate any specific challenges. The manager of Hospital 2 explicitly noted: “The process of digitalizing medical services in our hospital faces several challenges that influence the efficiency of implementing and using IT systems. One of the main challenges is the need to expand IT infrastructure (…). Another challenge is that current legislation still requires the existence of physical medical documents. The integration of different IT systems (…) and the high costs for equipment and software require sustainable financing solutions. Similarly, Hospital 11 highlighted that “the reasons hindering the digitalization process are financial (lack of funds) as well as the absence of specialized personnel to implement these solutions.” These accounts confirm that insufficient infrastructure, legislative constraints, interoperability issues, and financial difficulties remain central challenges across multiple institutions.

## 4. Discussion

**Data Triangulation**

Perceptions of healthcare digitalization in Romania are heterogeneous across stakeholders. While patient reports indicate frequent engagement with available digital services and perceived improvements in the service experience, managerial accounts, by contrast, suggest modest digital maturity and systems oriented primarily toward internal operations. The results that follow juxtapose these perspectives to identify areas of alignment and mismatch across contexts, with attention to sectoral and regional variation where relevant. Findings are presented as descriptive snapshots of current practice rather than causal estimates.

The data from the patient questionnaire indicate a high usage rate of digital services (81.6%), focused on administrative functionalities such as appointments, access to results, and payments (Figure 2). Their openness is clearly demonstrated through the word cloud analysis of patient priorities (Figure 7), which reveals a strong interest in key digital health initiatives, including expansion of telemedicine services, development of electronic health records, optimization of medical appointment systems, strengthening of family medicine, enhancement of cybersecurity, and integration of artificial intelligence.

However, the actual use of digital services is predominantly oriented toward administrative functionalities, which contribute to a more efficient and predictable patient experience. These services are perceived as useful and easy to integrate into daily routines, confirming findings in the literature that adoption is higher when benefits are clear, direct, and immediate [11,46,47]. 

In contrast, advanced digital services such as continuous monitoring of physiological parameters, mental health apps, or interaction with AI-based systems are significantly less utilized. For instance, only 11.2% of respondents had used mental health apps, and 8% participated in online support communities (Figure 2). This reluctance toward psycho-emotional or self-management functionalities reflects both a lack of trust in such technologies and their weak integration into the therapeutic pathway. This phenomenon may be explained by the absence of clear privacy policies, the lack of recommendations from healthcare professionals, and poor communication about the real benefits of these solutions [47].

A significant systemic issue is the limited use of telemedicine as a means of expanding access to healthcare services outside one’s place of residence. Only 33.6% of patients reported direct benefits in this regard, while 48% had never used such services. These findings underscore a major limitation in realizing the potential of telemedicine to enhance equity in healthcare. Non-use and abandonment are influenced by both individual factors (such as attitudes toward technology or digital literacy levels) and service-related factors (such as usability issues or lack of relevant content) [48].

Another indicator of low engagement is the reduced level of digital feedback: only 12.8% of patients provided feedback or evaluations of the digital services used. This result reflects a cultural passivity in the patient–technology relationship, possibly influenced by the absence of clear feedback mechanisms, concerns over privacy, or the perception that patient opinions are not valued. The literature emphasizes that active patient involvement in the design and evaluation of digital services is essential for increasing acceptance, and participatory methods can significantly contribute to shaping solutions that are tailored to real needs [49].

However, the managers’ perspective does not reflect this level of usage. In all 15 analyzed hospitals, IT systems are dedicated exclusively to internal use, with no platforms allowing patient access to their own data. Hospital managers’ responses indicate that EMR and EHR are perceived as synonymous, reflecting the reality of current systems that operate only at the organizational level, without interoperability or user-centered functionalities. Consequently, patient data remain fragmented and inaccessible, while the national EHR (DES) is still non-functional, leaving EHR an aspirational rather than a practical concept. Telemedicine is consistently used in only 13.3% of hospitals, and digital feedback mechanisms and personalized apps are entirely absent. Thus, although patients report relatively frequent usage, managers show that digital services are implemented in a fragmented manner and not oriented toward the patient, highlighting a significant gap between user perception and institutional reality. Moreover, patients demonstrate a clear perception of existing disparities between healthcare service providers in Romania, with 75.2% believing there are significant differences in digitalization levels between the public and private healthcare sectors. The word cloud analyzing patient concerns about health digitalization (Figure 6) captures this disparity perception, highlighting issues such as digital exclusion, inequalities in access to technology, and concerns about vulnerable populations or those in areas with limited infrastructure. This dichotomy reflects patients’ nuanced understanding of the current healthcare landscape, where they simultaneously embrace digital innovation while recognizing the uneven distribution of technological advancement across different healthcare providers.

An integrated analysis of the perceived impact of digitalization reveals both theoretical alignments and institutional divergences. According to patient data, digitalization is positively perceived in terms of access (65.6%), communication, and service quality, aligning with Blasiak et al. (2022) and Tițan et al. (2023) [50,51]. However, 28% adopt a neutral stance on access, an aspect that may be explained by infrastructural deficits, public–private disparities, and low digital literacy [8]. The results suggest that, although the overall use of digital services is not significantly associated with quality perception (Table 1), certain digital functionalities clearly have a favorable impact. The data support the hypothesis that digitalization of medical services significantly contributes to increased user satisfaction, especially by facilitating access, streamlining processes, and integrating modern communication channels. This finding reflects several important contextual factors: patients evaluate digital services against their baseline experiences within the local healthcare landscape rather than international benchmarks, making even incremental digitalization represent meaningful progress for populations historically underserved by technological solutions. The relative scarcity of digital healthcare tools may enhance their perceived value, with patients recognizing them as premium services that significantly improve their care experience compared to conventional alternatives.

Managers confirm these structural limitations: no hospital provides patients access to their own digital records, and interoperability between hospitals is practically nonexistent (93.3%). Therefore, patients’ perceptions regarding access and satisfaction (validation of Hypotheses H1 and H2) are not supported by the technical and functional realities in hospitals. The lack of inter-institutional connectivity highlights the fragmentation of the digital infrastructure and poses a major obstacle to ensuring continuity of care and facilitating a coherent medical pathway for patients.

Technical issues and usability difficulties reported by patients (over 28%) are correlated with the lack of digital literacy, and data security concerns (40.8%) confirm issues raised in the literature [52,53]. Managers cite the same barriers: outdated infrastructure (8 out of 15 responses), lack of digital competencies (4 responses), interoperability challenges, and limitations in national systems (5 responses). These constraints validate patient concerns and highlight the absence of a systemic digital strategy. Comparative analysis further shows that only a few hospitals reported investment projects, mainly aimed at IT infrastructure and hardware (e.g., Hospital 2). Such initiatives reflect more a process of digitization and automation of administrative flows, rather than genuine digitalization capable of transforming clinical services and improving patient access and experience.

Regarding the ethical dimensions of digital healthcare implementation, our findings reveal a complex patient perspective that encompasses multiple interconnected concerns about data protection, therapeutic relationships, and healthcare equity. The prominence of data security and confidentiality as the leading perceived risk (Figure 5), coupled with frequent mentions of medical data confidentiality risks in open-ended responses (Figure 6), demonstrates that patients are aware of privacy vulnerabilities inherent in digital health systems. These concerns are intrinsically linked to broader apprehensions about the transformation of the doctor-patient relationship, as evidenced by 22.4% of respondents expressing concerns about detachment or impersonality in digital services, and 32.8% reporting difficulties in symptom communication through digital platforms. The preference for face-to-face interactions and lack of trust in digital systems (Figure 4) further underscore patients’ reservations about whether digital platforms can adequately facilitate the nuanced communication necessary for proper informed consent and therapeutic alliance formation. Importantly, patients demonstrated sophisticated understanding of broader ethical implications, particularly regarding digital exclusion of vulnerable populations and those in areas with limited infrastructure (Figure 6), suggesting recognition that healthcare digitalization may paradoxically exacerbate existing inequalities while simultaneously offering potential benefits, which is a widely recognized fact in the scientific literature [54]. These interconnected concerns highlight the critical need for robust ethical frameworks that address not only technical data protection measures but also preserve the fundamental humanistic elements of healthcare delivery and ensure equitable access to digital health innovations.

From a conceptual perspective, these findings align with Donabedian’s framework (2005) [25]: at the structure level, infrastructure remains fragmented and insufficient; at the process level, digital tools are confined to administrative functions, with advanced clinical applications underutilized; and at the outcome level, the impact on access, equity, and quality remains low. International benchmarks such as the EGDI [27] and the Digital Decade eHealth Indicator [28] confirm that Romania lacks institutional mechanisms to adapt and apply maturity indicators at the level of public hospitals. Some managers (e.g., Hospitals 4 and 14) perceive digitalization merely as basic administrative computerization, without reference to maturity standards or alignment with international benchmarks, which explains why digital transformation remains at an early stage. This situation can be attributed to several systemic factors: the absence of a coherent national digital health strategy, reliance on fragmented and short-term projects often dependent on external funding, limited investment capacity of public hospitals, and persistent legislative constraints that still prioritize paper-based documentation. In addition, weak coordination between central authorities and healthcare institutions has prevented the development of a unified framework for assessing and monitoring digital maturity. Together, these gaps explain why digitalization initiatives remain superficial, unevenly distributed, and insufficient to produce measurable clinical or organizational outcomes.

Patients show openness toward the future of digitalization (87.2%), with priority directions aligned with the National Strategy 2023–2030 (e.g., electronic health records, AI, telemedicine) [55]. This convergence presents a strategic opportunity, but it depends on the system’s ability to deliver functional and equitable services [56,57] indicator. Managerial data confirm, however, a lack of digital maturity: 53.3% of hospitals do not run any digitalization projects. Only 6.7% have a partial form of interoperability, and telemedicine is suboptimally implemented and used (daily use in only 13.3% of cases). In these conditions, the potential benefits of digitalization remain largely unexploited and insufficiently felt in medical practice. As highlighted by the literature on change management ([43,58,59]), successful digital transformation depends not only on technology but also on organizational and cultural factors: stakeholder involvement, development of digital competencies, adaptive leadership, and fostering an innovation-oriented culture. Digitalization should therefore be understood not just as technology adoption, but as a broader process of redefining organizational mindsets and healthcare practices.

Data triangulation reveals a marked contrast between patients’ perceptions (optimism, satisfaction) and organizational reality (internally focused digitalization, lack of access, transparency, or coherent strategy). Hypothesis H1 is only partially confirmed: although patients report favorable perceptions of quality, the statistical analyses revealed a weak, inconsistent, and even negative relationship for certain digital services, suggesting that the use of these services raises expectations that are not always met. Hypothesis H2, however, is robustly confirmed: patient satisfaction is strongly influenced by tangible benefits (particularly easier access and online scheduling), although some functionalities may generate dissatisfaction when actual performance falls short of expectations. The integration of both perspectives highlights the urgent need for systemic reform focused on the patient, interoperability, and digital inclusion.

***Barriers to the Implementation of Digital Health Services in Romania.*** The study highlights several systemic barriers hindering the effective implementation of digital health, consistent with international literature:

- infrastructure deficiencies—outdated IT equipment, insufficient server capacity, and lack of mobile devices limit the stability, scalability, and advanced functions (e.g., real-time data exchange, telemonitoring) of digital platforms;
- low digital literacy among healthcare staff—resistance to new technologies, lack of structured training, and difficulties integrating digital tasks into daily workflows reduce adoption and consistent use of digital tools;
- policy and funding gaps—absence of a coherent national digital health strategy and reliance on fragmented, project-based initiatives result in unequal levels of digital maturity; limited funding constrains technology acquisition, maintenance, and integration;
- data security and privacy concerns—both patients and managers expressed worries about sensitive medical information and the lack of robust cybersecurity infrastructure and standardized secure data-sharing protocols. Trust in data protection mechanisms is seen as critical for adoption [52].
- low levels of patient empowerment and insufficient health literacy—many patients lack the knowledge, confidence, and skills required to effectively use digital tools (such as telemedicine platforms or electronic health records), which limits adoption and reduces the perceived benefits of digitalization.

Addressing these barriers requires an integrated strategy with sustained investments in infrastructure, comprehensive digital skills training for health professionals, coherent policy and funding frameworks, and internationally aligned data protection standards. Equally important, interventions should aim to strengthen patient empowerment and health literacy, ensuring that users can meaningfully engage with digital health services. Without such systemic interventions, the benefits of digital transformation risk being unevenly distributed, exacerbating disparities in access and quality of healthcare. In this regard, the set of standards developed in Supplementary File S3 (Reference VII—Digital Transformation Management) offers a practical framework that could be integrated into the next accreditation cycle or serve as a benchmark for excellence in digitalization. Developed in line with the ANMCS accreditation framework, which is currently in its third cycle, Reference VII fills an existing gap by introducing six themes, nine standards, and twenty criteria with their associated requirements. While in the current edition digital aspects are only addressed transversally (e.g., in risk management or patient relations), Reference VII provides a systematic structure and dedicated indicators for evaluating digital maturity. Covering domains such as infrastructure, interoperability, patient digital access, telemedicine, cybersecurity, competencies, and strategic management, these standards adapt international practices (JCI, ISQua, NABH) into actionable tools for Romanian hospitals. Their adoption would strengthen institutional accountability and accelerate the shift toward a smart, interoperable, and patient-centered health system. Ultimately, Romania’s digital maturity remains fragmented, and progress requires a systemic approach built on clear standards, monitored indicators, and sustained cultural and organizational transformation.

***Theoretical Implications.*** The research contributes significantly at the theoretical level by triangulating qualitative and quantitative data, offering an integrated view of digital health. Hypothesis H1 is only partially supported: certain digital services (e-prescriptions, mobile applications, medication delivery) are paradoxically linked to less favorable perceptions, a finding consistent with managers’ reports of systemic and organizational barriers. By contrast, Hypothesis H2 is robustly validated: patient satisfaction is strongly driven by tangible benefits, particularly easier access and online appointment scheduling. Together, the triangulated evidence underscores that digitalization enhances quality and satisfaction only when technology is aligned with institutional capacity and patient-centered design.

***Organizational and Practical Implications.*** Qualitative data from hospital managers reveal an institutional reality marked by systemic limitations: most hospitals use closed IT systems, designed exclusively for internal use, with no real patient access to medical data. Interoperability is absent in most cases, and telemedicine solutions either do not exist or are marginally used. The electronic health record is perceived as non-functional, with patient data remaining fragmented and inaccessible from outside the institution. These qualitative findings reinforce Hypothesis H1, showing that the perception of improved quality is linked not only to patient use of digital services but also to the institution’s ability to provide them in an integrated, functional, and transparent manner.

***Relevance for Public Policy.*** Both patients and managers identify converging priorities: the need for digitalization in family medicine, expansion of electronic health records, telemedicine functionalities, and use of artificial intelligence. These directions align with the National Health Strategy 2023–2030, suggesting a window of opportunity for coherent and participatory policies. At the same time, the lack of interoperability, absence of patient access to personal data, and significant disparities between the public and private sectors point to the urgent need for strategic investment and structural reform in digital infrastructure. The implementation of an integrated public policy in the field of digital health requires the coordinated involvement of key stakeholders such as the Ministry of Health, the National Authority for Quality Management in Health (ANMCS), regional public health authorities, hospital managers, IT solution providers, and professional and patient organizations. Priority actions should include: adopting unified national standards for the collection and interoperability of medical data, developing a coherent legislative framework for data governance, ensuring dedicated funding for interoperability and telemedicine projects, and providing continuous training for healthcare professionals in digital skills. These measures would accelerate the transition toward an intelligent, equitable, and patient-centered healthcare system. Without uniform, accessible, and patient-centered digitalization, a true digital transformation of Romania’s healthcare system cannot occur. This requires an integrated vision, coherent public policies, functional interoperability, continuous training, and digital equity.

***Limitations and Future Research Directions.*** The study is influenced by the characteristics of the sample, which consists predominantly of urban respondents with high levels of formal education, an aspect that may limit the generalization of conclusions. The invitations were sent by electronic means (e-mail, whatsapp groups) and the survey was carried out online using Google forms, so the sample includes patients who already use digital technologies and electronic communication means, possibly leaving out several segments of the society who are not familiar or do not have easy access to these technologies. Moreover, the qualitative analysis was focused on a relatively small number of institutions, which requires caution when extrapolating results to the healthcare system as a whole. In addition, the statistical associations for Hypothesis H1 were weak and somewhat sensitive to methodological specifications (e.g., data restructuring, multiple comparison corrections). This is not unexpected given the relatively small sample size (N = 125) in relation to the number of predictors, which limits statistical power. Accordingly, these findings should be interpreted with caution and replicated on larger, more diverse samples.

Despite the limited number of responses, particularly for the qualitative component, we consider that the study provides valuable insights specific to the current Romanian context, as well as relevant indications regarding existing barriers and stakeholder expectations. For the qualitative dataset, thematic saturation was assessed and deemed to have been reached based on the responses received, in line with established methodological guidelines [60].

To deepen the understanding of what drives the success or failure of digitalization, future research could expand the qualitative component through semi-structured interviews with medical staff, managers, and decision-makers, focusing on organizational barriers and the dynamics of technology adoption. A comparative analysis between public and private hospitals regarding digital maturity and patient perceptions of service quality would also be of interest. Another relevant direction would be to evaluate the long-term impact of digital initiatives on medical human resources, institutional efficiency, and equity in access to care. Such studies could help develop predictive models regarding the relationship between digital maturity and patient satisfaction, offering strong empirical foundations for public policy in the e-health domain.

## 5. Conclusions

This study investigated Romanian patients’ perceptions of medical service digitalization by integrating quantitative and qualitative methods. The objective was to investigate the extent to which digitalization in Romania addresses the current needs of patients, specifically testing whether digital technologies improve service quality and operational efficiency (H1) and enhance patient satisfaction and care access (H2). Findings indicate that H1 is only partially supported, as the association between technology use and perceived quality is weak, inconsistent, and in some cases negative, suggesting that digital tools may raise expectations that are not always met. By contrast, H2 is robustly validated: patient satisfaction is strongly associated with tangible benefits, particularly easier access and online appointment scheduling. The results also reveal a critical misalignment: patients express optimism toward digitalization, while hospitals report low digital maturity, with closed IT systems, poor interoperability, and limited telemedicine use. This gap highlights the urgent need for a systemic and coordinated digital health strategy that combines institutional capacity-building with patient-centered solutions. Priority measures include the adoption of national data standards, coherent governance legislation, dedicated funding for interoperability and telemedicine, and continuous digital skills training. To avoid reducing digitalization to administrative tasks, such initiatives must be linked to organizational change and cultural adaptation. Importantly, the set of proposed standards detailed in Supplementary File S3 can serve both as a practical reference for the next accreditation cycle and as a benchmark for excellence in healthcare digitalization.

## Figures and Tables

**Table 1:** Ordinal logistic regression results for the association between use of digital medical technologies (Q7) and perceived service quality (Q8). Odds ratios (95% confidence intervals) represent the change in odds of reporting one-level higher perceived quality when comparing users to non-users of each technology.

Digital Medical Technologies	Odds Ratio (95% CI) for a One-Level Increase in Perceived Quality When Comparing Users to Non-Users	*p* Value
Electronic prescription	0.23 (0.08–0.64)	0.005
Mobile health apps	0.23 (0.06–0.82)	0.024
Medication delivery	0.34 (0.11–1.0)	0.056
Online payment	3.0 (0.90–10)	0.073
Online access to test results	2.2 (0.86–5.5)	0.1
Platforms for medical education	3.0 (0.79–12)	0.108
Digital feedback	4.5 (0.69–30)	0.117
Wearables	3.2 (0.59–17)	0.178
Virtual consultations/telemedicine	1.9 (0.58–6.3)	0.292
Online appointments	1.6 (0.60–4.1)	0.363
Online support communities	0.48 (0.08–2.9)	0.419
ChatBots	0.61 (0.13–2.8)	0.532
Electronic medical record	1.2 (0.30–4.7)	0.817
Mental health apps	0.96 (0.20–4.6)	0.959

**Table 2:** Ordinal logistic regression results for the association between use of digital medical technologies (Q7) and patient satisfaction with digital medical services (Q10). Odds ratios (95% confidence intervals) represent the change in odds of reporting one higher level of satisfaction when comparing users to non-users of each technology.

Digital Medical Technologies	Odds Ratio (95% Confidence Interval) for a One-Level Increase in Satisfaction When Comparing Users to Non-Users	*p* Value
Online appointments	5.1 (1.9–13)	0.001
Medication delivery	0.25 (0.09–0.74)	0.013
Platforms for medical education	3.6 (0.92–14)	0.065
Online access to test results	2.1 (0.85–5.2)	0.105
Mobile health apps	0.38 (0.11–1.2)	0.107
Digital feedback	2.6 (0.48–14)	0.266
Virtual consultations/telemedicine	1.9 (0.60–6.2)	0.269
Wearables	2.3 (0.48–11)	0.297
Electronic prescription	0.65 (0.26–1.6)	0.361
Mental health apps	0.58 (0.13–2.6)	0.483
Online payment	1.3 (0.42–4.3)	0.625
Electronic medical record	0.84 (0.23–3.1)	0.791
Online support communities	0.81 (0.15–4.5)	0.805
ChatBots	1.0 (0.23–4.6)	0.982

**Table 3:** Ordinal logistic regression results for the association between perceived benefits of digitalization (Q9) and patient satisfaction with digital medical services (Q10). Odds ratios (95% confidence intervals) represent the change in odds of reporting one higher level of satisfaction when comparing users to non-users of each technology.

Predictor	Odds Ratio (95% Confidence Interval) for a One-Level Increase in Satisfaction Among Respondents Who Endorsed a Specific Benefit, Compared to Those Who Did Not	*p*-Value
Easier access to medical services	5.12 (2.11–12.4)	0
Greater convenience	2.16 (0.77–6.02)	0.143
Easier access to medical information	1.65 (0.74–3.70)	0.221
Reduced medical risks	1.64 (0.65–4.12)	0.296
Less bureaucracy/more transparency	0.61 (0.22–1.67)	0.34
Personalized care	1.83 (0.43–7.74)	0.41
Lower costs	0.79 (0.29–2.13)	0.635
Time savings	1.16 (0.49–2.76)	0.741
Better health monitoring	1.09 (0.34–3.44)	0.89

**Table 4:** Ordinal logistic regression results for the association between patient satisfaction with digital medical services (Q10) and a set of predictors derived from the use of specific digital medical technologies (Q7), the perceived benefits of digitalization (Q9), level of communication with providers (Q11), and the platforms employed for digital communication with physicians (Q12). Odds ratios (95% confidence intervals) represent the change in odds of reporting one level higher level of satisfaction when comparing users to non-users of each technology.

Predictor	Odds Ratio (95% Confidence Interval) for a One-Level Increase in Satisfaction Among Respondents Who Endorsed a Specific Benefit, Compared to Those Who Did Not	*p* Value
Easier access to medical services	8.17 (2.52–26.5)	0
Medication delivery	0.11 (0.03–0.41)	0.001
Online appointments	4.36 (1.28–14.8)	0.019
Less bureaucracy/more transparency	0.26 (0.07–0.89)	0.033
Platforms for medical education	4.71 (0.93–23.8)	0.061
Reduced medical risks	2.79 (0.82–9.46)	0.099
Virtual consultations/telemedicine	3.56 (0.78–16.3)	0.102
Easier access to medical information	2.00 (0.72–5.59)	0.185
Electronic prescription	0.52 (0.18–1.54)	0.238
Digital feedback	3.67 (0.41–33.1)	0.246
Online access to test results	1.81 (0.61–5.42)	0.287
Mobile health apps	0.48 (0.11–2.13)	0.332
Greater convenience	1.86 (0.53–6.49)	0.334
Online support communities	0.40 (0.04–4.09)	0.441
Better health monitoring	0.66 (0.13–3.36)	0.62
Mental health apps	1.68 (0.21–13.4)	0.624
Dedicated apps	0.45 (0.02–11.3)	0.626
Lower costs	0.79 (0.23–2.74)	0.71
Electronic medical record	1.33 (0.26–6.85)	0.73
E-mail	1.70 (0.06–49.7)	0.757
Wearables	1.32 (0.18–9.46)	0.781
WhatsApp/Messenger	0.65 (0.03–15.0)	0.788
Telephone	0.66 (0.03–14.6)	0.791
Online payment	0.92 (0.23–3.66)	0.909
Personalized care	1.08 (0.20–5.80)	0.928
Time savings	0.96 (0.32–2.85)	0.941
ChatBots	0.97 (0.17–5.64)	0.97

## Data Availability

The data presented in this study are available on request from the corresponding author.

## Supplementary Materials

The following supporting information can be downloaded at: https://www.mdpi.com/article/10.3390/healthcare13202549/s1, File S1: patient questionnaire questions; File S2: managers questionnaire questions; File S3: Reference VII—Digital Transformation Management. References [61,62,63,64,65,66,67,68,69,70,71,72,73,74,75,76,77] are cited in Supplementary Materials.

## Abbreviations

The following abbreviations are used in this manuscript:

H	Hypothesis
Q	Question
AI	Artificial Intelligence
ANMCS	National Authority for Quality Management in Health
CNAS	National Health Insurance House
EHRs	electronic health records
EMRs	electronic medical records
EU	European Union
IoT	Internet of Things
IT	Information Technology
SIUI	Integrated Single Information System
