# Supplementary material for: Digital Transformation of Medical Services in Romania: Does the Healthcare System Meet the Current Needs of Patients?
Source: Healthcare (Basel). 2025 Oct 10;13(20):2549. doi: 10.3390/healthcare13202549 (PMC12563242; doi:10.3390/healthcare13202549)
Supplement: Supplementary file 1 [file healthcare-13-02549-s001.zip › healthcare-3798329-supplementary.pdf]

Supplementary File S1 Mixed-Methods Questionnaire For Patients

I agree to participate in this study:

Yes (I wish to continue)
No (I wish to end the questionnaire)

I consent to the processing of personal data provided through this questionnaire, with the information being anonymized and used exclusively for research purposes during the study period:

Yes (I wish to continue)
No (I wish to end the questionnaire)

1) **Age (<18): …**

2) **Gender:**
    Male
    Female
    Prefer not to answer

3) **Residence Area:**
    Urban
    Rural
    Bucharest

4) **Education Level:**
    No formal education
    Compulsory education (10 grades)
    High school, without Baccalaureate
    High school, with Baccalaureate
    Post-secondary education
    University degree

Postgraduate degree

5) **Occupational Status:**

Student/Pupil
Employed
Freelancer
Entrepreneur
Retired
Unemployed

6) **Have you used digital medical services?** (e.g., online appointments, virtual consultations, health apps on your phone, etc.)

Yes
No

7) **What types of digital medical services have you used?**

○ Virtual consultations / Telemedicine (including second opinions)
○ Electronic prescriptions
○ Online appointments
○ Online payment for medical services
○ Online access to medical test results
○ Medication delivery services
○ Health mobile apps (e.g., monitoring blood sugar, heart rate, blood pressure, menstrual cycle; medication reminders, etc.)
○ Mental health apps (therapy, guided meditation, etc.)
○ Chatbots (virtual assistants for general questions/advice)
○ Online support community platforms
○ Platforms for medical education
○ Wearable devices (e.g., smartwatches, rings, patches for collecting medical data)
○ Electronic medical records with health history
○ Digital feedback (including after in-person consultations or hospital stays)
○ I have not used any

8) **How do you evaluate the impact of adopting digital technologies on the quality of healthcare services?**

Significantly improved
Slightly improved
Unchanged
Slightly worsened
Significantly worsened

9) In what ways has the quality of medical services been improved through digitalization?

○ Easier access to medical services (through online appointments, telemedicine, accessing results, etc.)
○ Easier access to medical information (through educational platforms or online communities)
○ Time savings
○ Reduced costs (e.g., travel expenses)
○ Increased convenience/comfort
○ Reduced medical risks (e.g., waiting in lines at the doctor during flu season; travel for fragile patients, etc.)
○ Reduced bureaucracy and increased transparency
○ Better monitoring of health status (for oneself or family members)
○ Personalized medical care (through wearable devices, tailored recommendations, etc.)
○ I don't know / Prefer not to answer

10) How has the adoption of digital technologies influenced your satisfaction with medical services?

Significant improvement
Moderate improvement
No change
Moderate decline
Significant decline

11) How do you think the digitalization of medical services has affected your communication with doctors (e.g., through telemedicine)?

Interpersonal communication is better (e.g., due to increased accessibility, psychological comfort, etc.)
Interpersonal communication is worse (e.g., lack of face-to-face interaction, difficulty expressing oneself, technological limitations, etc.)

I don’t think digitalization has influenced this aspect

12) Which platforms do you use the most for digital communication with doctors?

WhatsApp / Text messages / Other messengers

Zoom / Google Meet / Microsoft Teams, etc.

Email

Phone calls

Platforms/apps specifically designed for this purpose (e.g., those of private networks)

13) From your perspective, what are the main barriers to using digital medical services?

- ○ Associated costs (smartphone/tablet, smartwatch, subscriptions, etc.)
- ○ Difficulties in using the internet or accessing medical platforms/apps
- ○ Technical issues (errors, poor internet connection, etc.)
- ○ Lack of trust in digital systems
- ○ Preference for face-to-face communication and physical interaction
- ○ Lack of promotion of these services (I’m unaware they exist / I don’t know the benefits)
- ○ I have not encountered any barriers

14) From your perspective, what are the main risks of digital medical services?

- ○ Risks related to data security and confidentiality
- ○ Dependence on technology (e.g., in case of malfunctions, data can be lost, consultations canceled, etc.)
- ○ Difficulties in effectively communicating symptoms
- ○ Feeling of detachment / Lack of trust
- ○ Risk of incorrect or incomplete diagnosis/treatment
- ○ Hidden costs for additional services

15) To what extent do you believe digital transformation has provided opportunities to improve access to healthcare services?

To a very great extent

To a great extent

Moderately

To a small extent

To a very small extent

16) Have you accessed medical services or specialists through telemedicine that were not available in your locality?

Yes

No

I don't know / Prefer not to answer

17) Do you wish for the development of digital services in the Romanian healthcare system?

Yes

No

Indifferent

18) Do you believe there are currently significant differences between the digitalization of services in the public versus private healthcare systems?

Yes

No

I don't know / Prefer not to answer

19) To what extent do you believe digital technologies have the potential to radically transform the healthcare system in the next 10 years?

To a very great extent

To a great extent

Moderately

To a small extent

To a very small extent

20) What are your concerns regarding digitalization in healthcare?

………………………………………………………………………………………

21) Which areas or aspects do you believe should be prioritized for digitalization in the healthcare system?

………………………………………………………………………………………

Supplementary File S2 Qualitative Questionnaire For Hospital Managers

What types of information systems are used in hospitals under ASSMB (Administration of Hospitals and Medical Services of Bucharest) for managing patient data and medical services?

Please specify the name of the system used (Hipocrate, InfoWord, or other relevant systems).

What is the purpose of each system mentioned?

(Examples of use: patient information management systems, appointment scheduling systems, medication inventory management systems, etc.)

Is there an integrated system that allows interoperability between different hospitals?

Please specify the type of system used and how it facilitates the exchange of information between hospitals (access to patient medical records from different hospitals, data transfer).

What telemedicine and teleassistance solutions are available in hospitals under ASSMB? What is the level of usage of these solutions?

What is the status of the use of electronic health records in hospitals under ASSMB?

Please specify if the electronic health record is actively used in hospitals and to what extent it contributes to improving the quality of patient care and the efficiency of medical processes.

What are the main challenges encountered in the process of digitalizing medical services in hospitals under ASSMB? What measures are planned or being implemented to overcome these obstacles?

Are there any ongoing projects in hospitals under ASSMB that focus on technology investments (e.g., implementing new information systems, upgrading IT infrastructure, developing telemedicine solutions, or training staff in the use of digital technologies)?

Please specify the types of projects, their objectives, the current stage of implementation, and the funding sources for these initiatives.

Supplementary File S3 Reference VII – Digital Transformation Management

Special Report No. 25/2024 of the European Court of Auditors shows that, although EU support for healthcare digitalization has been effective, the use of funds has been hindered by administrative procedures and the lack of unified monitoring mechanisms. Digital maturity is assessed through the eGovernment Benchmark and the Digital Decade eHealth indicator; however, issues of accuracy and methodology have been reported [61]. At the same time, the *Digital Government Review of Romania* highlights the country's persistent challenges in consolidating digital governance and the need for alignment with international standards [10]. These orientations, which acknowledge the importance of digitalization and establish development directions, are also reflected in international accreditation standards such as the **ISQua Guidelines (2025)** [62], **Joint Commission International (2024)** [63], and the **NABH Digital Health Standards (2023)** [64], which already integrate the digital component into the quality assessment of healthcare services. In addition, national policy documents [65,66], the **ENISA Report (2024)** [67], and the **ISO/IEC 27799:2016** [68] standard strengthen the framework for cybersecurity and health data protection. Therefore, Romanian hospitals must explicitly include the digital dimension in performance evaluation, as its absence would prevent them from meeting end-user requirements and risk compromising the quality of healthcare services.

The **National Authority of Quality Management in Health (ANMCS)** is responsible for ensuring and continuously improving the quality of health services and patient safety through the standardization and accreditation of healthcare facilities. Its accreditation standards, internationally recognized by ISQua, are structured into thematic "references," each comprising criteria, requirements, and indicators mandatory for hospitals [69]. At present, **ANMCS is developing the standards for the third accreditation cycle**, structured into six references and updated themes; however, none explicitly addresses digital transformation, despite its essential role in the organization and quality of medical services [70]. The standards are not yet in their final form, but the proposed themes confirm the absence of a dedicated, unified approach to digitalization. [71]

To address this gap, we propose **Reference VII – Digital Transformation Management**, a framework for evaluating hospitals' digital maturity, directly linked to patient access, service quality, and clinical safety. The modular structure of this reference enables gradual and comparable evaluations across hospitals, while ensuring alignment with international standards. Thus, it becomes a flexible instrument applicable to all types of healthcare facilities, regardless of size, profile, or legal status.

Methodology for the Development of the Proposed Standards:
The standards proposed under Reference VII – Digital Transformation Management were developed through a structured methodology combining legislative analysis, international benchmarking, and contextual adaptation to the Romanian accreditation framework. The drafting process was based on:

- The national accreditation framework provided by the Hospital Accreditation Standards Manual [69] and the updated thematic directions for the third accreditation cycle [70], which formed the structural foundation of the proposed standards.

- Analysis of national regulations and strategic documents, including: Order no. 5.813/2024 of the Ministry of Health and the Special Telecommunications Service and Order no. 56/2024 [72,73], Digitalization Guide [66], and National Artificial Intelligence Strategy [65], and the National Health Strategy 2023–2030 [54].

- European and international policy frameworks, such as: Digital Decade 2030 Policy Programme [74], eHealth Digital Decade Indicators [75], and Digital Decade Country Report: Romania [28], complemented by the UN E-Government Development Index [76] and the UN E-Government Survey 2024 [27], and the Global Digital Health Monitor [77].

- International accreditation and evaluation practices, including the ISQua Guidelines (2025) [62], Joint Commission International standards (2024) [63], and NABH Digital Health Standards (2023) [64].

- Cybersecurity and data protection frameworks, illustrated by the ENISA State of Cybersecurity in the Union Report [67] and ISO/IEC 27799:2016 [68].

- Audit and evaluation evidence, notably Special Report No. 25/2024 of the European Court of Auditors, which emphasized both the effectiveness of EU support for healthcare digitalization and the persistent challenges related to fund allocation and monitoring mechanisms [61].

- Methodological contributions, including the Digital Decade eHealth Indicators Development – Final Report , which supported the operationalization of digital health indicators and informed the development of measurable criteria.

This methodology ensures that the proposed standards are both internationally aligned and locally applicable, providing hospitals with a coherent framework for self-assessment and continuous improvement in digital maturity.

Reference VII – *Digital Transformation Management* (R7) follows a modular structure consistent with the model used in previous ANMCS editions. It comprises six main themes, each addressing a key functional domain of digitalization in healthcare institutions. Every

theme is supported by specific standards, codified in the 7.x.y format, where 7 designates R7, *x* indicates the theme (T1–T6), and *y* represents the standard within that theme.

This structure enables a clear, stepwise, and comparable assessment of digital maturity across hospitals and accreditation cycles, ensuring:

- comprehensive evaluation of digital maturity in all hospital functions;
- alignment with international standards (e.g., NABH, ISQua, JCI, ISO);
- integration of European indicators (eHealth Decade, eGovernment Benchmark);
- adaptability to all hospital types, regardless of size, profile, or legal status.

Overall, Reference VII includes **6 fundamental themes, 9 standards, and 20 criteria**, reflecting the core domains where digitalization has a major impact (Tables 5-10).

**Theme 1**<br>Digital Infrastructure and Interoperability	
**Standard 7.1.1.** The digital infrastructure is functional, secure, and adapted to the needs of the healthcare unit	
**Criterion: 7.1.1.1** The hospital has the infrastructure necessary to support medical activities through modern and integrated information systems.	**Requirement**<br>✔ The network is supported by functional backup solutions and alternative power sources (UPS/generator) for critical equipment;<br>✔ The digital platform is accessible via browser and mobile application, compatible with at least two major browsers;<br>✔ An integrated information system is routinely used across all departments;<br>✔ Generation of a unique patient identifier, with automatic mechanisms to prevent duplicates;<br>✔ Synchronization of records created offline, without data loss;<br>✔ The hospital uses a digital system for managing admissions and allocating patients to wards and beds;<br>✔ Management of admissions in the absence of available beds through digital triage and deferred scheduling;

	✔ Complete digital registration of emergency cases, including when admission is not required;<br>✔ All medical data are associated with the unique patient identity, with the possibility of controlled sharing with other institutions;<br>✔ A designated IT officer is in place, with maintenance ensured internally and/or through external contracts;<br>✔ Documented and active maintenance plans are in place and periodically monitored;
**Criterion: 7.1.1.2** The hospital has the infrastructure necessary to support administrative activities through modern and integrated information systems.	**Requirement:**<br>✔ Existence of an IT module for human resources management (timekeeping, job descriptions, evaluations);<br>✔ Use of an application or other digital system for economic management: budget, accounting, inventory, procurement;<br>✔ Management and archiving of administrative documents is carried out digitally or digitized (with electronic signature where applicable);<br>✔ Administrative workflows are automated: internal requests, approvals, notifications.
**Criterion:** 7.1.1.3 The hospital uses digital systems for managing paraclinical investigations (laboratory and imaging).	**Requirement:**<br>✔ Assignment of a unique digital ID for each sample, linked to the patient;<br>✔ LIS integrated with laboratory equipment for automatic transmission of results;<br>✔ The hospital has a documented procedure for recording results from rapid tests and non-integrated POCT analyzers, as well as a plan for digitally integrating these results into the unit's information system;

	✔ Patient digital access to laboratory reports via portal/application;<br>✔ Use of RIS/PACS for creation, validation, and digital issuance of reports;<br>✔ Digital archiving in DICOM format with full traceability;<br>✔ Patient digital access to radiology and laboratory reports via portal/application;<br>✔ Secure export and sharing of reports between departments and with other institutions under conditions of information security.
**Standard 7.1.2.** The hospital ensures the functional interoperability of internal and external information systems.	

**Criterion 7.1.2.1** The hospital ensures interoperability of its information system with external platforms and internal applications.	✔ The hospital is functionally and continuously connected to national platforms (SIUI, DES), with automatic and bidirectional transmissions;<br>✔ International integration standards are used (e.g., HL7, FHIR, DICOM, CDA) to ensure system compatibility;<br>✔ Internal applications are interconnected, allowing efficient sharing of clinical and administrative data across all departments;<br>✔ The hospital has protocols and agreements for interoperability with external institutions and the capacity to share patient data (with hospitals, outpatient clinics, pharmacies, etc.) under secure and regulated conditions;<br>✔ Externally transmitted data are encrypted, authenticated, and subject to IT auditing;<br>✔ The hospital integrates into its internal digital record results originating outside the unit (e.g., laboratory tests or imaging performed by partners);<br>✔ Functional user logs are maintained, and updated documentation exists for all internal and external connections;<br>✔ Discharge and inter-institutional transfers are managed digitally, with complete, secure documentation accessible to the receiving hospital as well as the patient;<br>✔ The hospital has the technical capacity to receive in real time clinical data and test results performed in pre-hospital settings, including from ambulances, prior to patient arrival;<br>✔ Medico-legal documentation is transmitted digitally and securely to the Institute of Forensic Medicine (IML) or other authorized institutions upon request.

Table 5. Theme 1 proposed within Reference VII, with its specific standards and criteria

**Theme 2**<br>Patient Digital Access

**Standard 7.2.1.** The hospital facilitates digital interaction with patients through secure access to medical data and active communication.	
**Criterion 7.2.1.1.** The hospital provides patients with secure digital access to the electronic health record.	**Requirement:**<br>✔ Existence of a secure patient portal and/or functional mobile application;<br>✔ The hospital's digital systems ensure secure user authentication through eID, OTP, or two-factor authentication (2FA), particularly for access to sensitive medical data and critical applications;<br>✔ Patient information and training on how to use the system (via guides, materials, or direct support);<br>✔ Retention of patient access logs to prevent external intrusion/security breaches;<br>✔ Easy patient access to medical documents (tests, medical letters, consultation records, hospitalizations, data generated in the Emergency Department) and integration with the EHR (once it becomes fully operational).
**Criterion 7.2.1.2.** The hospital actively communicates with and educates patients through digital means.	**Requirement:**<br>✔ General information about hospital activities is available online (e.g., schedule, locations, departments, contact details);<br>✔ Educational materials for patients are published on the hospital's digital platform (website or application);<br>✔ Accreditations and certifications are displayed publicly in digital format;<br>✔ Administrative and informational notifications are transmitted digitally to patients (newsletter, SMS, application);<br>✔ The digital platform allows online scheduling for consultations, investigations, or procedures;<br>✔ Patients can select the desired specialty and physician, as well as the available time slot;

	✔ The system notifies the patient regarding confirmation, rescheduling, or cancellation;<br>✔ The attending physician and/or medical staff can view their digital schedule (including patient history).
**Standard 7.2.2.** The hospital ensures equitable digital accessibility for patients belonging to vulnerable groups.	
**Criterion 7.2.2.1.** The hospital's digital systems are adapted to allow functional access for vulnerable patients, under conditions of equity, security, and support.	**Requirement:**<br>✔ The digital platform complies with web accessibility standards (at least WCAG 2.1, Level AA);<br>✔ New digital services (e.g., online scheduling, access to medical records) are tested prior to implementation with patients from vulnerable groups (elderly, persons with disabilities, individuals without internet access);<br>✔ Alternative access channels are available (human assistance, telephone, support points);<br>✔ A documented internal procedure exists regarding vulnerable patients' access, establishing identification criteria, support measures, and clear responsibilities, under conditions of equity, security, and support.

Table 6. Theme 2 proposed within Reference VII, with its specific standards and criteria

**Theme 3**<br>Telemedicine and e-Health	
**Standard 7.3.1.** The hospital employs telemedicine and e-health solutions for the provision and optimization of medical services, in accordance with applicable regulations.	
**Criterion 7.3.1.1.** The hospital provides telemedicine services (remote consultations, monitoring, evaluation) for eligible specialties.	**Requirement:**<br>✔ Existence of a functional telemedicine platform that complies with personal data security regulations;<br>✔ Consultations performed through telemedicine are documented and

	integrated (updated) into the patient's record;<br>✔ Training of medical staff in the use of the platform.
**Criterion 7.3.1.2.** The hospital facilitates the use of e-health technologies for information, self-assessment, and scheduling.	**Requirement:**<br>✔ Patient access to mobile/web applications for appointments and communication;<br>✔ Integrated digital feedback systems;<br>✔ Demonstrated functionality of applications through indicators (e.g., usage rate);<br>✔ Secure digital communication channels.

Table 7. Theme 3 proposed within Reference VII, with its specific standards and criteria

**Theme 4**<br>Cybersecurity and Data Protection	
**Standard 7.4.1.** The hospital implements technical and organizational measures to ensure the cybersecurity of its information systems.	
**Criterion 7.4.1.1.** The hospital has technical measures in place for cybersecurity protection.	**Requirements**<br>✔ The hospital employs cybersecurity solutions such as firewalls, antivirus software, and IDS/IPS systems to protect IT infrastructure.<br>✔ Multi-factor authentication (2FA) is implemented for access to sensitive systems.<br>✔ Sensitive data are encrypted both in transit (network) and at rest (servers, databases).<br>✔ IT systems maintain activity logs, and access is constantly monitored.<br>✔ Backups are performed automatically at regular intervals and are periodically tested for restoration.
**Criterion 7.4.1.2.** The hospital applies active policies and procedures for managing cybersecurity risks.	**Requirements**<br>✔ The hospital has a formal IT security policy, approved by management and updated annually.

	✔ The digital system allows secure single sign-on (SSO) for authorized users.<br>✔ A cyber incident response plan is in place, including measures for business continuity.<br>✔ An IT team or designated officer is appointed with clearly defined cybersecurity responsibilities.<br>✔ The hospital maintains a functional mechanism for reporting and escalating incidents to the Data Protection Officer (DPO) and relevant authorities.
**Standard 7.4.2.** The hospital ensures the protection of personal data and the resilience of IT infrastructure through technical and organizational measures, while safeguarding patient rights.	
**Criterion 7.4.2.1.** The hospital implements technical and organizational measures for personal data protection.	**Requirements**<br>✔ The hospital has an internal policy on personal data protection, approved and reviewed annually, in compliance with applicable legislation (e.g., GDPR, Law 190/2018).<br>✔ A Data Protection Officer (DPO) is appointed, registered with the competent authority, and assigned updated responsibilities.<br>✔ The digital or physical system allows for the collection of informed, specific, freely given, and revocable patient consent.<br>✔ A documented procedure exists for revoking consent and informing the patient about the legal basis, duration, and purpose of data processing.<br>✔ Patients can access, rectify, or delete their data through mechanisms compliant with GDPR rights.
**Criterion 7.4.2.2.** The hospital manages digital infrastructure and IT risks to ensure continuity, confidentiality, and data security.	**Requirements**<br>✔ Comprehensive digital inventory of IT infrastructure (hardware, network, software) and documented plans for maintenance and periodic updates.<br>✔ Regular IT audits conducted internally or by an external provider.<br>✔ Disaster recovery plans for both clinical and administrative applications.

	✔ An IT incident reporting system with full traceability of resolutions.<br>✔ Active protection mechanisms: multi-factor authentication (2FA), encryption in transit and at rest, firewalls, antivirus, IDS/IPS.<br>✔ Automatic termination of inactive sessions and prohibition of using personal email accounts for professional purposes.

Table 8. Theme 4 proposed within Reference VII, with its specific standards and criteria

**Theme 5**<br>Digital Competencies of Healthcare Staff	
**Standard 7.5.1.** The hospital develops and supports staff digital competencies through training, assessment, and the use of digital tools.	
**Criterion 7.5.1.1.** Staff are trained and periodically assessed in the digital competencies required for their activities.	**Requirements**<br>✔ An annual digital training program is in place for medical, administrative, and technical staff.<br>✔ Mandatory participation of new employees in digital induction sessions.<br>✔ Use of digital platforms for training, feedback, and record-keeping of courses and certifications.<br>✔ Periodic evaluation of digital competencies, with integration of results into the performance management process.
**Criterion 7.5.1.2.** The hospital uses digital tools for human resource management and supports continuous professional development	**Requirements**<br>✔ An active digital platform for recruitment, onboarding, contractual notifications, and staff offboarding.<br>✔ Performance evaluation is digitalized and accessible to both the employee and the manager.<br>✔ Staff have secure access to digital educational resources (e-learning platforms, journals, guidelines).<br>✔ A support system is in place to assist staff with difficulties in using digital equipment or applications.

Table 9. Theme 5 proposed within Reference VII, with its specific standards and criteria

**Theme 6**<br>Strategic Digital Management	
**Standard 7.6.1.** The hospital uses digital systems to monitor quality, safety, and patient satisfaction.	
**Criterion 7.6.1.1.** Patient feedback is collected and analyzed digitally and used in strategic planning.	**Requirements**<br>✔ A digital system for collecting feedback (patient portal, app, terminal, etc.);<br>✔ Standardized forms with options for anonymous or personalized completion;<br>✔ Centralization and periodic analysis of data, presented to management for performance improvement;<br>✔ Traceability of implemented improvement measures;<br>✔ Automatic generation of reports on patient satisfaction.
**Criterion 7.6.1.2.** Digital systems support monitoring of medical care quality and safety.	**Requirements**<br>✔ Digital system for tracking healthcare-associated infections;<br>✔ Digital access to antibiotic protocols;<br>✔ Digital recording of medication errors and automatic alerts;<br>✔ System-assisted identification of vulnerable patients through automatic completion of care plans upon admission (e.g., infections, pressure ulcers, falls) and monitoring throughout hospitalization;<br>✔ Periodic analysis of data and integration into quality indicators.
**Criterion 7.6.1.3.** Medication management is digitally supported to prevent medication errors.	**Requirements**<br>✔ Digital inventory of medicines and consumables, with periodic updates;<br>✔ Digital records for controlled substances (e.g., narcotics, psychotropics);<br>✔ Digital recording of medication dispensing and distribution to departments;<br>✔ System notifications for expired medicines;<br>✔ Approved medication formulary digitally accessible to prescribing physicians.

**Criterion 7.6.1.4.** The hospital evaluates the impact and costs of digital systems.	**Requirements**<br>✔ A documented cost–benefit analysis process prior to implementing new digital solutions;<br>✔ Identification, evaluation, and management of risks related to interoperability and affected workflows;<br>✔ Assessment of the impact on staff, patients, and clinical safety;<br>✔ Periodic adjustment of digital systems based on feedback, errors, or observed dysfunctions.
**Criterion 7.6.1.5.** The hospital governs the use of Artificial Intelligence (AI) systems through policies compliant with applicable legislation and the ethical principles of digital health governance.	**Requirements**<br>✔ The hospital has an internal policy or procedure regarding the use of AI systems in medical or administrative activities.<br>✔ AI systems are used in compliance with applicable national and European legislation, including the European AI Act and national strategic frameworks, while respecting principles of ethics, safety, and data protection.<br>✔ An internal approval and monitoring mechanism is in place for introducing AI systems, including risk assessment, evaluation of equity impacts, and unintended consequences.<br>✔ Institutional policy clearly defines human responsibility in decision-making, preventing full delegation of decisions to AI.<br>✔ Patients are transparently informed when interacting with AI systems, and informed consent includes this aspect.<br>✔ The hospital periodically monitors the performance, impact, and potential risks of AI systems, including possible discriminatory effects or algorithmic errors.<br>✔ Corrective measures are provided, including internal audits and reporting mechanisms for AI-related incidents.

Table 10. Theme 6 proposed within Reference VII, with its specific standards and criteria
